# Comparative analyses of structural features and scaffold diversity for purchasable compound libraries

**DOI:** 10.1186/s13321-017-0212-4

**Published:** 2017-04-21

**Authors:** Jun Shang, Huiyong Sun, Hui Liu, Fu Chen, Sheng Tian, Peichen Pan, Dan Li, Dexin Kong, Tingjun Hou

**Affiliations:** 1 State Key Laboratory of Agricultural Microbiology and Agricultural Bioinformatics Key Laboratory of Hubei Province, College of Informatics, Huazhong Agricultural University, Wuhan, 430070 Hubei China; 2 College of Pharmaceutical Sciences, Zhejiang University, Hangzhou, 310058 Zhejiang China; 3 State Key Lab of CAD&CG, Zhejiang University, Hangzhou, 310058 Zhejiang China; 4 College of Pharmaceutical Sciences, Soochow University, Suzhou, 215021 Jiangsu China

**Keywords:** Scaffold diversity, TCMCD, Scaffold Tree, Tree Map, SAR Map

## Abstract

Large purchasable screening libraries of small molecules afforded by commercial vendors are indispensable sources for virtual screening (VS). Selecting an optimal screening library for a specific VS campaign is quite important to improve the success rates and avoid wasting resources in later experimental phases. Analysis of the structural features and molecular diversity for different screening libraries can provide valuable information to the decision making process when selecting screening libraries for VS. In this study, the structural features and scaffold diversity of eleven purchasable screening libraries and Traditional Chinese Medicine Compound Database (TCMCD) were analyzed and compared. Their scaffold diversity represented by the Murcko frameworks and Level 1 scaffolds was characterized by the scaffold counts and cumulative scaffold frequency plots, and visualized by Tree Maps and SAR Maps. The analysis demonstrates that, based on the standardized subsets with similar molecular weight distributions, Chembridge, ChemicalBlock, Mucle, TCMCD and VitasM are more structurally diverse than the others. Compared with all purchasable screening libraries, TCMCD has the highest structural complexity indeed but more conservative molecular scaffolds. Moreover, we found that some representative scaffolds were important components of drug candidates against different drug targets, such as kinases and guanosine-binding protein coupled receptors, and therefore the molecules containing pharmacologically important scaffolds found in screening libraries might be potential inhibitors against the relevant targets. This study may provide valuable perspective on which purchasable compound libraries are better for you to screen.

**Electronic supplementary material:**

The online version of this article (doi:10.1186/s13321-017-0212-4) contains supplementary material, which is available to authorized users.

**Graphical abstract:**
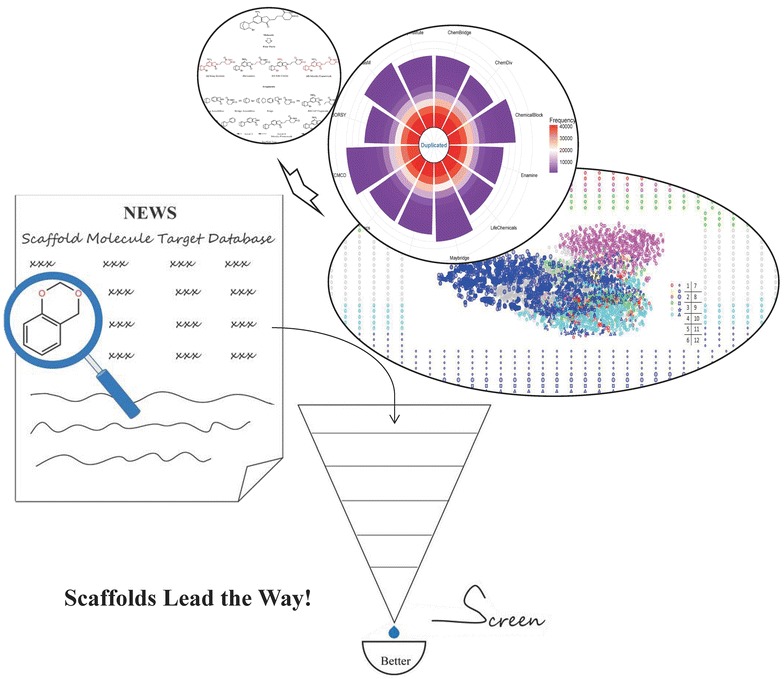
Selecting diverse compound libraries with scaffold analyses.

## Background

Virtual screening (VS) based on a variety of ligand-based or structure-based drug design approaches, such as property-based or drug-likeness rules, quantitative structure–activity relationship (QSAR) models, pharmacophore hypotheses, molecular docking, has become a powerful way to find hits in drug discovery. Certainly, screening libraries of small molecules with 2-D or 3-D structures are indispensable sources for VS campaigns. For example, the number of purchasable molecules collected in the ZINC database increases from ~0.73 million in 2005 to over 100 million in 2015 [1]. For the 176 vendors deposited in ZINC15, 37 offer more than 100,000 compounds and 9 offer more than 1 million compounds. This highlights the progress in synthesis of organic chemistry and tremendous demand of this market. In most VS applications, it is more practical and time effective to screen a compound library provided by a specific vendor rather than screen all compound libraries collected by ZINC. Certainly, the distributions of physiochemical properties, structural features and scaffold diversity of purchasable compound libraries afforded by different vendors should be different [2]. Therefore, an important question may be raised: *which library should be used for VS*? In order to answer this question, we need to have a deep understanding of the intrinsic features of each purchasable compound library and the difference among them.

**Fig. 1:**
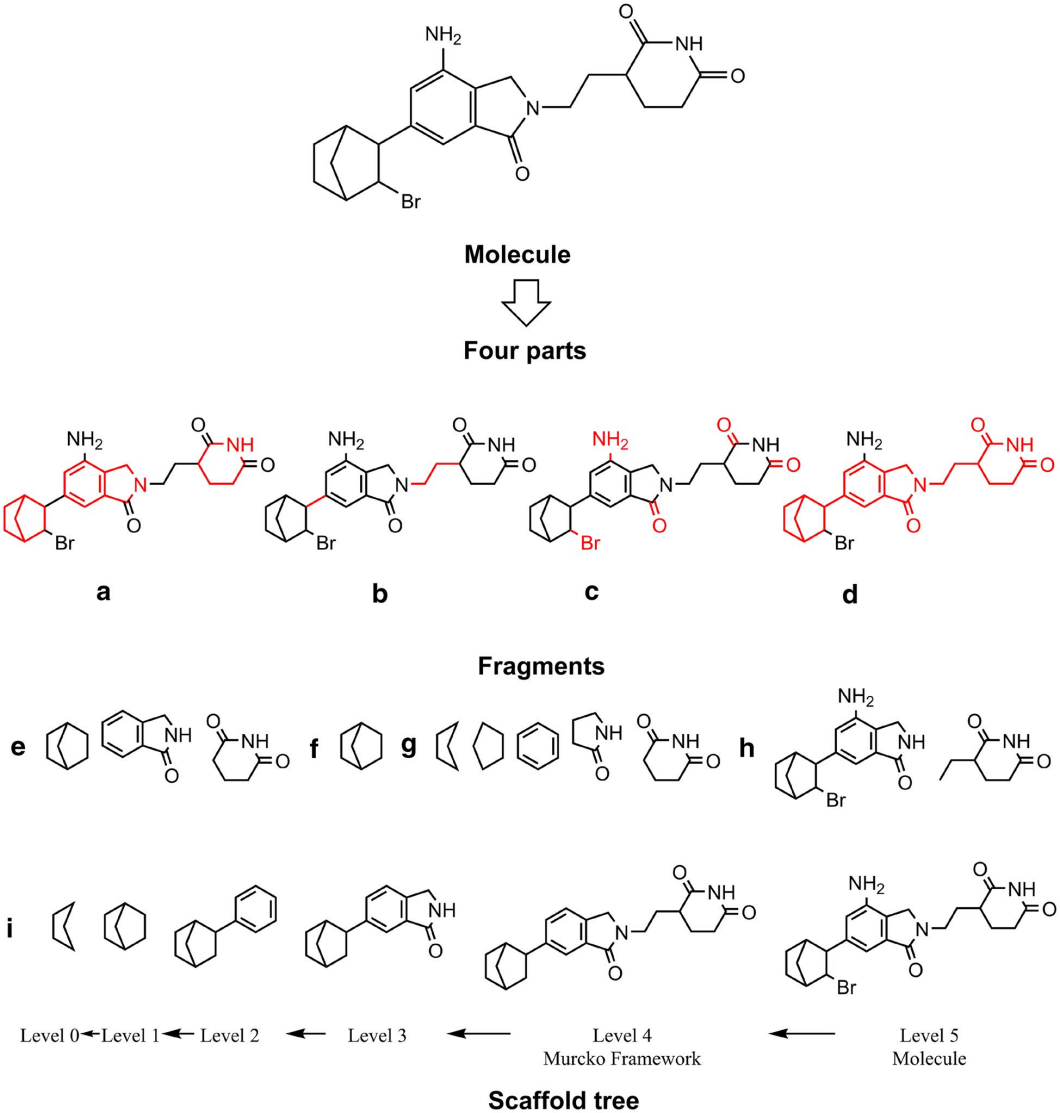
Definitions of different types of fragments in a molecule: **a** ring systems, **b** linkers, **c** side chains and **d** Murcko framework, **e** ring assemblies, **f** bridge assemblies, **g** rings, **h** RECAP fragments and **i** scaffold tree

As we know, the properties of a molecule are determined by its structure, and therefore similar structures tend to bear similar properties according to the similarity principle [3]. Thus, the chemical space of a compound library should be examined by molecular structures, especially chemical scaffolds, which has a huge impact on the success rate in biological screenings [4]. The scaffold of a molecule can be described by different ways. The most traditional way to define a scaffold is the Markush structure proposed by Markush [5]. Markush structures are usually used in patent applications to define chemical series [6], but they may be too generic to highlight the important structural features essential for pharmaceutical activity. Another scaffold representation is the Murcko framework proposed by Bemis and Murcko [7]. This method employs a more systematical way to dissect a molecule into four parts: ring systems (Fig. 1a), linkers (Fig. 1b), side chains (Fig. 1c), and the Murcko framework (Fig. 1d) that is the union of ring systems and linkers in a molecule. Lewell et al. [8] described a more chemically meaningful presentation of molecular structures, namely “RECAP” (retrosynthetic combinatorial analysis procedure), which cleaves molecules at bonds based on 11 predefined bond cleavage rules derived from common chemical reactions. As an example shown in Fig. 1h, the molecule is dissected into two parts at the bond linked by nitrogen and carbon. Therefore, analysis of compound libraries by using the RECAP representation may be a good way to explore the synthetic feasibility of a molecule.

Based on the Murcko framework, Schuffenhauer et al. [9] proposed a more complicated and systematical methodology, called Scaffold Tree (ST), to describe the ring systems arranged in a hierarchical tree, which iteratively prunes rings one by one based on a set of prioritization rules until only one ring remains. The structural hierarchies of each molecule in a Scaffold Tree are numbered numerically from Level 0 (the single remaining ring usually) to Level *n* (the original molecule) (Fig. 1i), and Level *n* − 1 is the Murcko framework. Owing to the systematic partition of molecular structures, the Scaffold Tree methodology has been employed in many scaffold diversity studies of compound libraries [10–12].

A number of studies have been reported to analyze and compare the chemical space and diversity of commercially available compound libraries in the last decade [13]. Krier et al. [14] evaluated the scaffold diversity of 17 commercially available screening collections with 2.4 million compounds by analyzing the maximum common substructures (MCS), and they grouped the commercial collections into different categories with low, medium and high scaffold diversity. However, the definition of MCS is arbitrary and data set dependent, and MCS may be not a general way to represent a large number of scaffolds. Langdon et al. [12] analyzed the structural diversity of 7 commercial compound libraries by using the Murcko frameworks and Scaffold Trees, and then visualized the scaffold space by using the Tree Maps software [15]. They found that there were some emblematical scaffolds in each library. Nevertheless, the libraries analyzed by Langdon et al. are rarely used in practical VS and the numbers of molecules in three libraries are even <10,000, and therefore the results may not be informative for drug design/discovery. With the rapid increase of the number of commercially available small molecules, analysis of the structural features and scaffold diversity for representative screening libraries is quite demanding.

In this study, the structural features and scaffold diversity of eleven commercially available screening libraries and Traditional Chinese Medicine compound database (TCMCD) were explored by analyzing seven fragment representations. All the selected commercial libraries have more than 50,000 compounds and have been widely used in VS. We aimed to find the difference of the structural features and scaffold diversity among these libraries. Tree Maps and SAR Maps [16] were used to visualize the distribution of the scaffolds based on the similarity of molecular fingerprints. Moreover, the underlying pharmacological characteristics, that is the potential targets of the molecules with the representative scaffolds, were also examined. We believe that our study will help the decision making process when selecting commercially available compound libraries for VS.

## Methods

### Preparation and standardization of libraries

The 11 large compound libraries deposited in ZINC15 were chosen in the analysis, and they are Mcule, Enamine, ChemDiv, VitasM, UORSY, ChemBridge, LifeChemicals, ZelinskyInstitute, Specs, ChemicalBlock and Maybridge. Mcule is the largest library in ZINC15, and it contains 4,922,295 molecules. The SDF files of the studied libraries were downloaded from the vendors’ websites (Additional file 1: File S1). TCMCD developed in our group was also included in this study, and it contains 57,809 molecules with molecular weight (MW) lower than 800, which are found in more than 5000 herbs used in traditional Chinese medicines (TCM) [17–19]. The basic information of the studied libraries is summarized in Table 1. Then, the molecules in all libraries were preprocessed by the following Pipeline Pilot protocol: fixing bad valence, filtering out inorganic molecules, adding hydrogens and removing duplicated molecules [20].

**Table 1:** Basic information of the 12 studied libraries

Databases^a^	Number^a^	Filtered^b^	Description^c^
Mcule	4,922,295	4,876,889	Large, individual service
Enamine	1,959,026	1,958,807	Lead-like, diverse
ChemDiv	1,741,807	1,741,603	Selected
VitasM	1,460,248	1,460,009	Novel compounds
UORSY	1,301,092	1,293,353	Original and unique
ChemBridge	1,064,558	1,064,425	Selected, derivatives
LifeChemicals	413,286	412,788	Selected
ZelinskyInstitute	381,214	379,048	No descriptions
Specs	212,404	212,332	Selected
ChemicalBlock	125,791	125,473	Selected, diverse
Maybridge	57,809	57,490	Highly diverse
TCMCD	54,206	54,138	Natural product

^a^Number of all molecules in each library

^b^Number of the molecules in each library after processed by different filters

^c^Simple description of the studied libraries

**Fig. 2:**
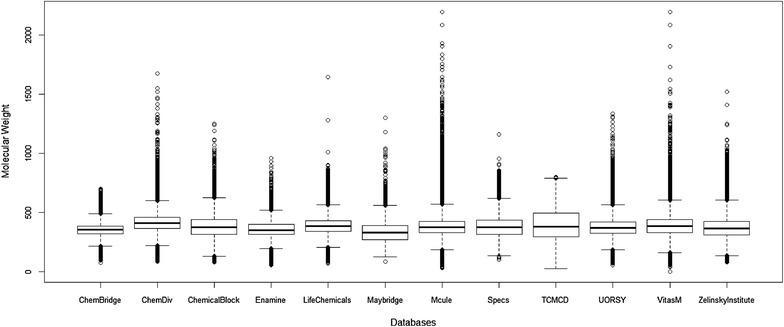
Box plots of the distributions of molecular weight for the 12 studied databases

The MW distributions of the studied libraries are shown in Fig. 2. It can be observed that ranges of MW for these libraries vary greatly. Then, we analyzed the MW distributions at an interval of 100 and found that the numbers of molecules in some intervals for different libraries are quite different. Molecules in the studied libraries with MW from 100 to 700 are highly overlapped. Thus the distributions of MW should be standardized in order to eliminate the influence of MW on scaffold analysis [21]. Eventually, based on the least number of molecules at each interval of 100 MW within the studied libraries, the same numbers of molecules were randomly selected at each interval for all libraries and then 12 new standardized subsets were generated. The standardized subsets have the equal numbers of molecules (41,071) and almost identical MW distributions ranging from 100 to 700. The following analyses were conducted based on the 12 standardized subsets.

### Generation of fragment presentations

A total of 7 fragment representations were used to characterize the structural features and scaffolds of molecules, and they are ring assemblies, bridge assemblies, rings, chain assemblies, Murcko frameworks [7], RECAP fragments [8], and Scaffold Tree [9].

The first five types of fragment representations were generated by using the *Generate Fragments* component in Pipeline Pilot 8.5 (PP 8.5) [20]. The RECAP fragments and Scaffold Tree for each molecule were generated by using the *sdfrag* command in MOE [22]. Owing to the lack of the original molecules in the Scaffold Tree provided by the *sdfrag* command, the missing original molecules were added to the SDF files of the Scaffold Tree using PP 8.5 (Additional file 1: File S1). The generation of the Scaffold Tree (from Level 1 to Level *n*) was accomplished in PP 8.5 by defining the fragments at different levels for each molecule. Eventually, the SDF files of these fragment representations were obtained (Additional file 1: File S1).

### Analyses of scaffold diversity

The scaffold diversity of each standardized dataset was characterized by the fragment counts and the cumulative scaffold frequency plots (CSFPs) or so called cyclic system retrieval (CSR) curves [23, 24]. The duplicated fragments were removed first, and the numbers of unique fragments for each dataset were counted for ring assemblies, bridge assemblies, rings, chain assemblies, Murcko frameworks, RECAP fragments and Levels 0–11 of Scaffold Tree, along with the numbers of molecules they represent (referred to as the scaffold frequency).

Then, the scaffolds were sorted by their scaffold frequency from the most to the least, and the cumulative percentage of scaffolds was computed as the cumulative scaffold frequency divided by the total number of molecules [12]. Similarly, percentages of unique fragments can also be calculated. Then, CSFPs with the number or the percentage of Murcko frameworks and Level 1 scaffolds, which may better represent the whole molecules than the other types of fragments, were generated. In each CSFP, PC50C was determined for each scaffold representation to quantify the distribution of molecules over scaffolds. PC50C was defined as the percentage of scaffolds that represent 50% of molecules in a library [14].

### Generation of Tree Maps

The Tree Maps methodology was employed to analyze the structural similarity of the Level 1 scaffolds by using the TreeMap software, which can highlight both the structural diversity of scaffolds and the distribution of compounds over scaffolds. Tree Maps has been used as a powerful tool to depict structure–activity relationships (SARs) and analyze scaffold diversity [25]. Different from traditional tree structure represented by a graph with the root node and children nodes from the top to the bottom, Tree Maps proposed by Shneiderman uses circles or rectangles in a 2D space-filling way to delegate a kind of property for a clustered dataset with clearly intuitive visualization [15]. Thus, one can visualize a hierarchical clustering map by organizing those clustered properties along with other features for a dataset, such as MW.

First, the unique Level 1 scaffolds were clustered by using the *cluster molecules* component in PP 8.5 based on the ECFP_4 (extensive-connectivity fingerprint 4) fingerprints [26–28]. According to Tian’s study [29] and our testing, although the clustering method is order dependent, the order dependency of the *cluster molecules* component did not have obvious effect on the clustering results. So, recentering the cluster center twice in a clustering protocol is enough. Then, the SDF file of the clustered scaffolds for each standardized dataset was converted into a text formatted file, which was used as the input of the TreeMap software [30] (Additional file 1: File S1). In each Tree Maps, scaffolds are represented by circles with gray perimeters. The area of each circle is proportional to the scaffold frequency, and the color of each small circle is related to the DTC (DistanceToClosest, i.e., the distance between the fragment and the cluster center) of fragments in each cluster. The lowest value of DTC for the Level 1 scaffolds of ChemBridge (DTC = 0) was colored in red, the highest value (DTC = 0.778) in deep green and the middle value in white. The highest values of DTC for the other databases were also around 0.8. The yellow labels in each Tree Maps were the order numbers of clusters.

### Generation of SAR Maps

SAR Maps generated by the DataMiner 1.6 software is usually used to organize high throughput screening (HTS) data into clusters of chemically similar molecules, which provides a good way for interactive analysis. This structural clustering allows identification of possible false negatives and false positives in the data when the colors in the map represent experimental activity values. The map can not only display the results effectively, but also provide a convenient way to access the chemical series presented by the maximum common structure (MCS) scaffolds. Along with SAR (structure–activity relationship) rules, and substructure- and property-based tools provided in DataMiner, the SAR Map is a powerful method assisting to make the best possible decision on which molecules should be studied further.

First, the cluster centers of the top 10 most frequently occurring clusters of the Level 1 Scaffolds observed in the Tree Maps for each standardized subset were defined as the queries to search the dataset by using the *Substructure Filter from File* component in PP 8.5. The 4816 identified records (i.e., original molecules) were saved into a SDF file (Additional file 1: File S1).

Then, the *Generate SAR Map* function in DataMiner 1.6 was used to generate the structure similarity maps, i.e. SAR Maps [16]. The K-dissimilarity Selection or OptiSim method [31–33] was used to select a diverse and representative samples from the original dataset based on the Tanimoto similarity distances calculated from the 2D UNITY structural fingerprints [34]. Because the SAR Map is not a simple plot of two variables, it does not have axes. For *N* compounds, the SAR Map is an optimal projection of the *N*-squared similarities within the points onto a two dimensional plot using the nonlinear mapping (NLM) projection method [35]. Singleton Radius and SAR Map Horizon are two critical parameters to control the map. The Singleton Radius represents a dissimilarity radius, which was set to 0.3. A singleton is a compound that does not have any nearest neighbor within a predefined radius, and it is regarded as a point in the hedge of the map. The SAR Map Horizon was also set to 0.3, which means that two points will be placed far apart if the dissimilarity between them is higher than the parameter value, but their distance is not in scale relative to the others’ on the map. Accordingly, molecules gathered on the map definitely characterizing much more similar compounds are more meaningful than those separated ones. Therefore, 40 denser areas or so called representative molecules were selected and shown with black dotted circles on the SAR Map. The similarity between molecules in each area and its central molecules were higher than 0.8 (including 0.8), and these representative molecules in an area were saved as a SDF file (Additional file 1: File S1). Then selected molecules from each circle were used as the queries to identify the similar molecules in the BindingDB database [36]. In similarity search, the structural similarity threshold for each query was adjusted to make sure that at least one similar compound could be found for each query, and the least similarity threshold was set to 0.6. Finally, the potential targets of 39 queries were assigned to those of the similar molecules found in BindingDB.

## Results and discussion

### Counts of fragments

For the 12 standardized subsets, the fragments based on seven types of fragment representations, including ring assemblies, bridge assemblies, rings, chain assemblies, Murcko frameworks, RECAP fragments and Scaffold Tree scaffolds, were generated. The total numbers of all and unique fragments are listed in Tables 2 and 3. Because the standardized subsets have the identical numbers of molecules (41,071) and approximately the same MW distributions, the impact of MW on the analysis of fragments can be eliminated and the counts of the dissected molecules (i.e. fragments) can be compared and analyzed directly.

**Table 2:** Numbers of the duplicated and non-duplicated ring assemblies (ra), bridge assemblies (b), rings (r), chain (c), Murcko framwork (m) and RECAP fragment (RECAP) for the 12 standardized datasets

Databases	Total number						Non-duplicated number					
	ra	b	r	c	m	RECAP	ra	b	r	c	m	RECAP
ChemBridge	105,467	964	125,082	514,422	41,024	493,990	1255	85	543	3450	25,788	107,898
ChemDiv	103,562	440	129,997	512,142	40,933	369,011	2021	69	784	3493	21,875	93,439
ChemicalBlock	96,236	1204	125,442	492,515	40,870	250,765	2355	106	888	3369	17,045	63,061
Enamine	99,387	496	117,219	474,170	40,832	496,594	1130	39	523	6002	26,870	94,869
LifeChemicals	103,421	431	128,421	493,056	40,973	370,651	1063	34	531	2603	20,276	68,912
Maybridge	94,063	577	110,054	461,415	40,841	264,327	1408	68	729	3543	15,242	53,852
Mcule	101,088	538	122,696	492,813	40,874	419,190	2144	75	812	5368	27,247	108,294
Specs	96,202	872	119,323	494,752	41,038	336,076	1889	82	832	3154	15,259	72,454
TCMCD	58,111	5793	127,355	466,842	39,192	702,520	8509	1351	1176	5962	12,941	104,631
UORSY	96,675	454	110,588	471,902	40,678	521,182	829	28	449	6120	21,491	91,776
VitasM	98,063	650	122,978	493,391	40,871	321,898	2132	64	839	3939	20,108	81,702
ZelinskyInstitute	96,430	1128	117,460	481,948	40,927	310,800	1533	72	669	3145	16,666	68,365

**Table 3:** Numbers of the duplicated and non-duplicated scaffolds at different levels of Scaffold Tree for the 12 standardized datasets

Level	ChemBridge	ChemDiv	ChemicalBlock	Enamine	LifeChemicals	Maybridge	Mcule	Specs	TCMCD	UORSY	VitasM	ZelinskyInstitute
*Duplicated scaffolds*												
0	41,022	40,930	40,861	40,832	40,973	40,841	40,873	41,038	39,138	40,677	40,868	40,923
1	41,019	40,918	40,856	40,820	40,964	40,836	40,869	41,029	39,122	40,665	40,860	40,919
2	38,267	38,499	37,846	36,963	38,566	36,138	37,559	37,377	34,703	35,902	37,428	37,036
3	27,397	29,625	26,445	24,095	28,565	21,634	26,333	23,746	25,632	21,767	25,862	23,242
4	11,992	14,686	12,640	9315	12,968	7045	11,871	10,770	14,752	7922	12,2345	9984
5	2652	3307	3471	2039	2802	1301	2726	2933	6650	1594	3263	2683
6	363	342	552	317	272	212	419	415	1715	196	606	421
7	47	24	54	32	10	19	60	48	306	26	90	61
8	24	5	5	2	4	2	11	2	43	4	6	6
9	2		1			2	2	1	9		2	1
10			1			1			6		1	
11									3			
*Non-duplicated scaffolds*												
0	594	716	871	571	467	713	801	822	1110	482	804	684
1	8997	7112	7789	11,283	6524	9406	10,992	8142	8083	10,632	8736	8504
2	26,560	20,385	20,145	29,034	20,080	22,645	28,041	21,155	14,158	26,941	22,236	22,384
3	24,810	24,129	19,642	22,850	22,258	18,434	24,178	18,086	14,822	20,433	20,848	18,597
4	11,604	13,948	11,114	9042	11,889	6623	11,542	9727	10,414	7808	11,301	9067
5	2606	3265	3293	1965	2731	1253	2673	2859	5723	1586	3103	2593
6	347	339	545	312	271	209	405	413	1587	194	585	416
7	31	23	54	32	9	19	60	48	298	26	90	61
8	24	5	5	2	4	2	11	2	43	4	6	6
9	2		1			2	2	1	9		2	1
10			1			1			6		1	
11									3			

Obviously, two kinds of fragments contain side chains, including chain assemblies (chains) and RECAP fragments. The percentages of molecules that do not have any ring in the standardized subsets were also calculated, and they are 0.12, 0.34, 0.51, 0.58, 0.24, 0.56, 0.48, 0.08, 4.71, 0.96, 0.49 and 0.36% for ChemBridge, ChemDiv, ChemicalBlock, Enamine, LifeChemicals, Maybridge, Mcule, Specs, TCMCD, UORSY, VitasM and ZelinskyInstitute, respectively. Among the studied libraries, TCMCD has the highest percentage of acyclic molecules (close to 2000), which is consistent with the results reported by Tian et al. [29]. However, the total number of chains in TCMCD is the least but one (466,842). More interestingly, TCMCD has 5962 unique chains, which are almost twice to those in ChemBridge (3450). Considering that the standardized subset of TCMCD has more acylic compounds, less chains while more unique chains, it appears that the chains in TCMCD are bigger or more complicated and diverse. Despite Maybridge has the fewest number of chains (461,415), which is similar to TCMCD, its number of unique chains (3543) is at the average level, which is still higher than those of ChemBridge (3450) and ChemDiv (3493). However, Chembridge and ChemDiv bear the top two numbers of chains (>510,000). Thus, the structures in Maybridge may be more diverse, which needs to be explored by other types of fragment representations. Among the studied libraries, UORSY and Enamine have more non-duplicated chain assemblies (6120 and 6002) than the others, suggesting that they have more diverse chains, which are two times higher than that of LifeChemicals (2603). Moreover, Mcule owns relatively high number of unique chains (5368).

Another fragment representation containing side chains is RECAP fragments, which are the building blocks for synthesizing molecules. As shown in Table 2, TCMCD has extremely high number of RECAP fragments (702,520), indicating that, on the average, synthesizing a compound in TCMCD needs more RECAP fragments than synthesizing a molecule in any other standardized subset. That is to say, synthesizing these compounds in TCMCD may be quite difficult. ChemBridge, Enamine and UORSY have relatively high numbers of RECAP fragments (~500,000), which are almost twice comparing with those of ChemicalBlock (250,765) and Maybridge (264,327). Therefore, it may be easier to synthesize the molecules in ChemicalBlock and Maybridge.

In the other five types of fragment presentations, three of them belong to ring systems, including rings, ring assemblies and bridge assemblies. The total numbers of rings for all libraries are quite close, and the biggest difference is found between Maybridge (110,054) and ChemDiv (129,997). Similarly, the total numbers of ring assemblies of these libraries are not quite different, but TCMCD is the only exception. The number of all ring assemblies in TCMCD (58,111) is significantly fewer than those in the other libraries, but quite interestingly, the number of the unique ring assemblies in TCMCD (1351) is quite higher than those in the other libraries. Different from rings and ring assemblies, bridge assemblies characterize contiguous ring systems sharing two or more bonds, and therefore they are also ring assemblies but more complicated. As shown in Table 2, the total number of the bridge assemblies in TCMCD (5793) is significantly higher than those in the other libraries. Although the total number of the simple ring systems in TCMCD is not quite high, its unique numbers of the rings and ring assemblies are much higher than those of the other libraries. In a word, TCMCD has more complicated and diverse ring systems. However, commercial libraries generally contain more simple rings instead of multiple ring systems, such as bridge assemblies. Herein, as a whole, ChemicalBlock and Specs have more unique ring systems, as shown in Table 2. Mcule and VitasM have relatively diverse ring systems, and Mcule also has relatively diverse chains. Enamine and UORSY have relatively high numbers of unique chains, but the numbers of their distinctive ring systems are so low. For LifeChemicals, both of the numbers of the unique chains and ring systems are quite low, suggesting that it has relatively low structural diversity.

The other two types of fragment presentations, Murcko frameworks and Scaffold Tree, characterize molecular scaffolds, and they can represent the whole structural features for compounds in a library. Murcko frameworks, which are the union of ring systems (Fig. 1a) and linkers (Fig. 1b), are usually used as the structural signatures of molecules. As shown in Table 2, the total numbers of the Murcko frameworks for all the standardized subsets except TCMCD do not have large difference, which may result from much more acylic molecules found in TCMCD, but those of the unique ones are quite different. The number of the unique Murcko frameworks for Mcule (27,247) is the highest, while that for TCMCD (12,941) is the lowest, highlighting the fact that the structures of natural compounds may be more conservative than those of the synthesized molecules in commercially available libraries. Other databases, such as ChemBridge and Enamine, also possess relatively high numbers of Murcko frameworks (25,788 and 26,870, respectively). However, as mentioned above, the diversity of the ring systems for Enamine is pretty low.

**Fig. 3:**
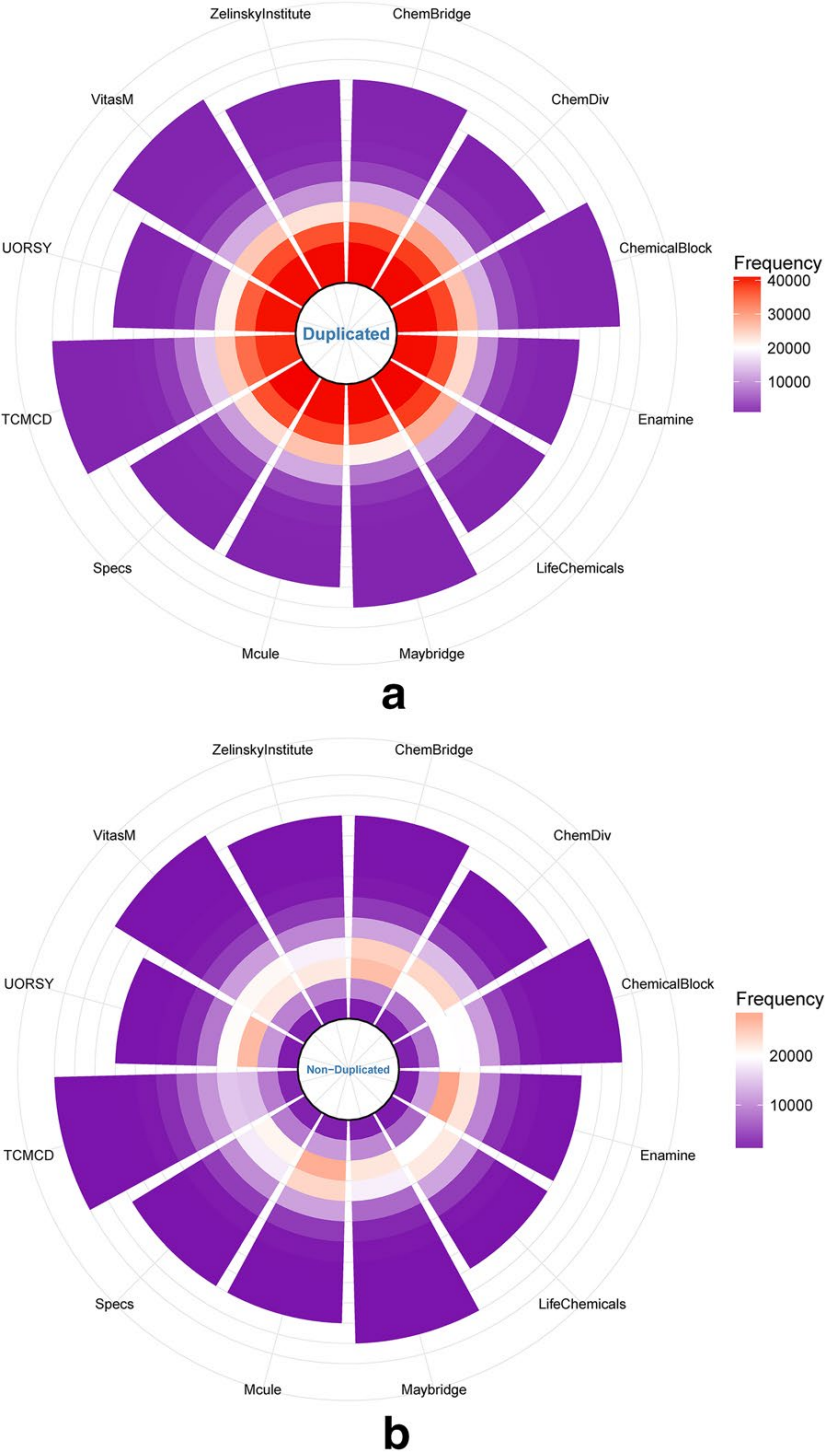
Rose maps for **a** the total numbers of the Scaffold Tree for the 12 datasets and **b** the non-duplicated numbers of the Scaffold Tree for the 12 datasets

Scaffold Tree is a series of rings by chopping molecules into smaller pieces. The numbers of the fragments found at 12 levels of the Scaffold Tree [9] are listed in Table 3. The number of the levels for each subset is determined by the maximum complexity of molecules. Obviously, the most complicated structures can be found in TCMCD. To better compare the differences at each level among the 12 libraries, rose maps were plotted and shown in Fig. 3. Twelve petals stand for the studied libraries, and the twelve layers on each petal depict Level 0 to Level 11 of the Scaffold Tree from inside to outside in turn. Frequencies of molecules can be easily identified and compared by colors. As shown in Fig. 3a, as the levels increase higher than Level 1, the numbers of the scaffolds decrease sharply. At the levels higher than Level 2, the numbers of the fragments for Maybridge, UORSY and ZelinskyInstitute are lower than those for the other libraries. For TCMCD, the numbers of the fragments at Levels 0–2 are relatively low, but those at Level 4 or higher are quite high. That is to say, TCMCD is rich in more complicated structures. In Fig. 3b, the numbers of the unique fragments at 12 levels show different trend comparing with those of all fragments at 12 levels. The numbers of the unique scaffolds at Level 0 are even much lower than those at Level 1, and the numbers of the unique scaffolds at Level 2 or 3 are the highest. It appears that ChemBridge, Enamine and Mcule have higher diversity at Levels 2 and 3 than the other libraries.

In summary, TCMCD contains much more complicated structures and its whole molecular scaffolds are more conservative than the commercial libraries. Generally speaking, at Levels 2 and 3, ChemBridge and Mcule show high structural diversity. At Level 5 or higher, ChemicalBlock, Specs and VitasM possess relatively high structural diversity, suggesting that these libraries contain more complicated structures. LifeChemicals has relatively high diversity for the Scaffolds at Levels 3 and 4, but has relatively low diversity for rings, ring assemblies and bridge assemblies (Table 2). Certainly, in order to characterize the structural diversity of the 12 studied libraries more clearly, further quantitative analyses are necessary.

### Cumulative scaffold frequency plots (CSFPs)

**Fig. 4:**
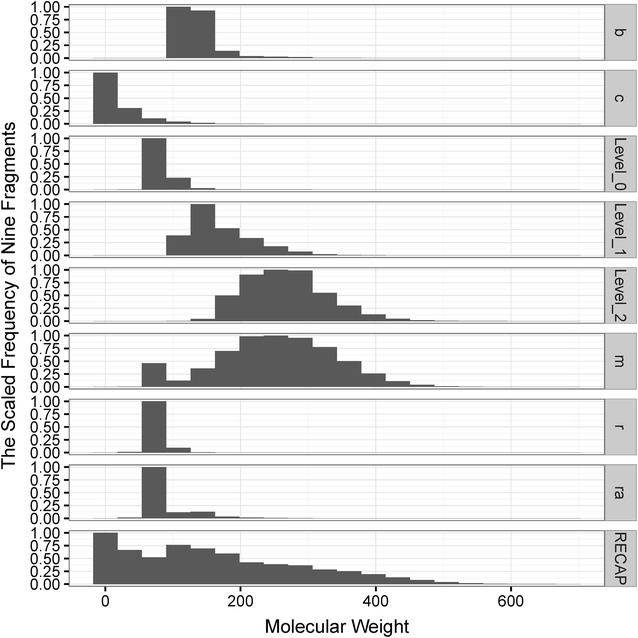
The scaled distributions of molecular weight for nine types of fragments found in the 12 datasets. Here, *b* represents bridge assemblies, *c* represents chain assemblies, Level_0, Level_1 and Level_2 represent Level 0, Level 1 and Level 2 of the Scaffold Tree, respectively, *m* represents Murcko frameworks, *r* represents rings, *ra* represents ring assemblies, and *RECAP* represents RECAP fragments

Among the seven types of fragment representations, which kind of representation is the best choice to characterize the diversity of molecules is a critical problem for us to solve. According to the result from Langdon et al. and Tian et al. [12, 29], considering the balance between structural complexity and diversity, Level 1 scaffolds and Murcko frameworks may be the best choice to represent the scaffolds for most molecules. Besides, the scaled distributions of MW of the fragments for the 12 libraries are shown in Fig. 4. Noticeably, the distributions of the Level 2 scaffolds and Murcko frameworks are quite similar. As for the RECAP fragments, many fragments are too small. Therefore, the Level 1 scaffolds and Murcko frameworks are better to represent the whole molecules, and they are used in the following analyses.

**Fig. 5:**
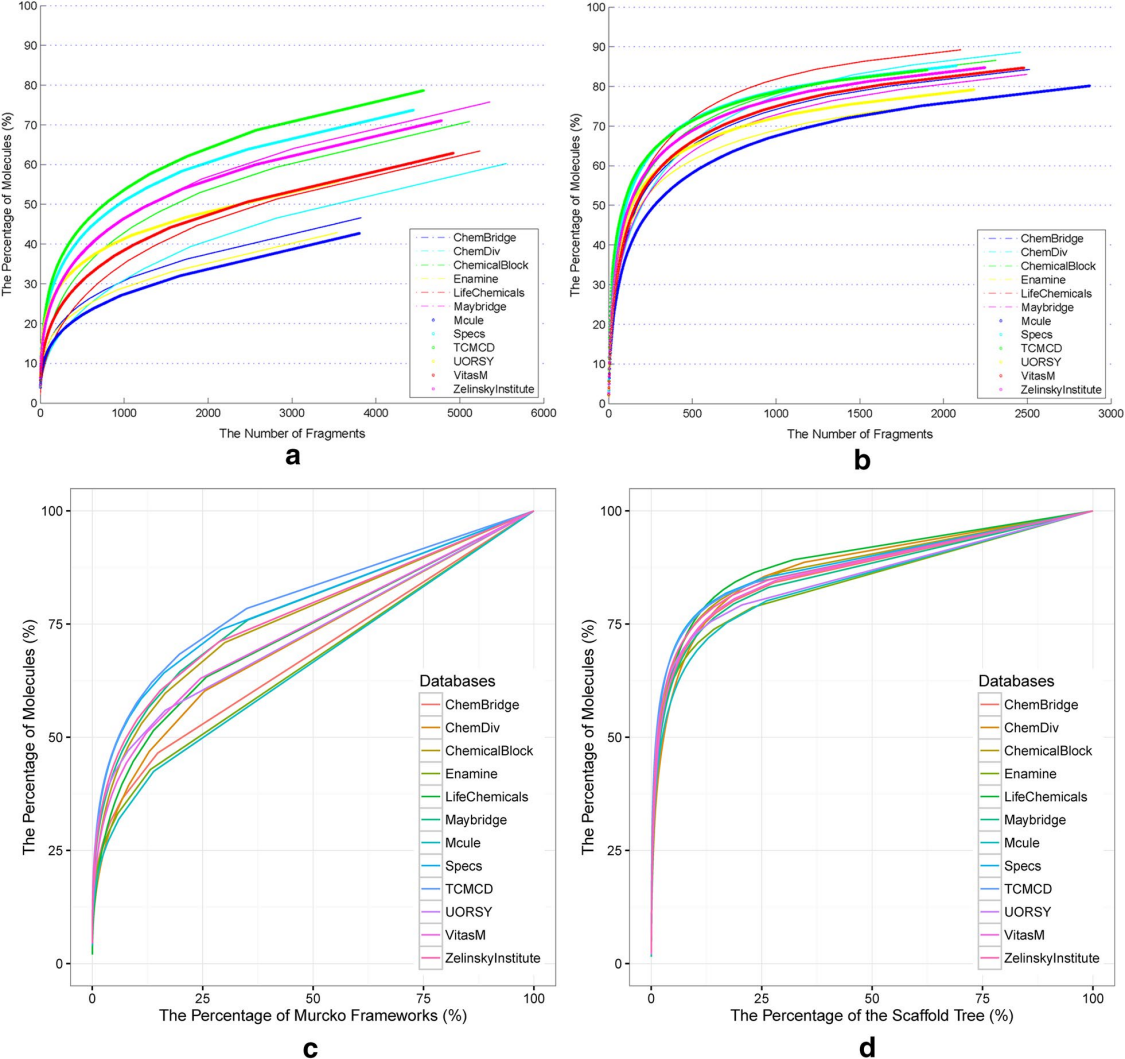
**a** Cumulative scaffold frequency curves of the Murcko frameworks, which is truncated at the point where the frequency of the fragment turns from 2 to 1, for the 12 dataset; **b** cumulative scaffold frequency curves of the Level 1 Scaffold Tree fragments, which is truncated at the point where the frequency of the fragment turns from 2 to 1, for the 12 datasets; **c** cumulative scaffold frequency plots (CSFPs) of the Murcko frameworks for the 12 datasets; **d** CSFPs of the Scaffold Tree fragments for the 12 datasets

The CSFP is a good way to analyze the diversity for large compound libraries. Scaffold frequencies are the number of molecules containing particular scaffolds, which can also be represented as the percentage of the compounds in a library. Similarly, the number of fragments can also be presented as the percentage of the total numbers as shown in Fig. 5. In Fig. 5a, b, curves were truncated at the point where the frequency of the fragment turns from 2 to 1 to compare them clearly considering the following lines are paralleled. By analyzing the CSFPs in these two figures roughly, we found that the slopes of the curves were different and the steeper curves suggested that the most frequently occurring scaffolds can be found in more molecules. For instance, the percentages of the molecules of the top ten frequently occurring Murcko frameworks are 7.625, 5.174, 7.042, 7.756, 4.540, 11.792, 6.938, 13.332, 11.015, 12.601, 8.710 and 11.005% for ChemBridge, ChemDiv, ChemicalBlock, Enamine, LifeChemicals, Maybridge, Mcule, Specs, TCMCD, UORSY, VitasM and ZelinskyInstitute, respectively.

However, different libraries do not have identical numbers of fragments, which may influence the direct comparison of the 12 standardized datasets. The information derived from the CSFPs in Fig. 5c, d can be roughly quantified by using the PC50C values, which is the percentage of scaffolds that represent 50% of molecules, as shown in Table 4. Accordingly, the higher the value of PC50C is, the more diverse the scaffolds of a database will be. As shown in Fig. 5c and Table 4, TCMCD reaches 50% at the lowest number of the Murcko frameworks, then Specs, Maybridge, Zelinsky Institute and ChemicalBlock. On the contrary, Mcule, Enamine and Chembridge do not reach 50% even the percentage of the most frequently occurring scaffolds become about 25% (Fig. 5a). According to the PC50C values of the Murcko frameworks for the 12 libraries (Table 4), the scaffold diversity of Mcule, Enamine, ChemBridge, ChemDiv, LifeChemicals, VitasM, UORSY, ChemicalBlock, Maybridge, ZelinskyInstitute, Specs and TCMCD can be ranked in a descending order. In Fig. 5d and Table 4, the rank of the Level 1 scaffolds, however, is a little bit different. The scaffold diversity of ChemDiv, Mcule, Maybridge, LifeChemicals, ChemBridge, VitasM, ChemicalBlock, Enamine, ZelinskyInstitute, UORSY, Specs and TCMCD are ranked in a descending order.

**Table 4:** PC50C values of the Murcko frameworks (Murcko) and Level 1 scaffolds for the 12 standardized datasets

Databases	PC50C	
	Murcko	Scaffold Tree
ChemBridge	21.38	1.92
ChemDiv	16.03	2.82
ChemicalBlock	9.42	1.68
Enamine	26.41	1.68
LifeChemicals	12.96	1.99
Maybridge	8.52	2.09
Mcule	27.36	2.49
Specs	6.15	1.28
TCMCD	5.92	1.11
UORSY	11.10	1.30
VitasM	11.85	1.86
ZelinskyInstitute	7.85	1.47

The scaffold diversity evaluated based on the Level 1 scaffolds and Murcko frameworks deliver similar overall trends. Three libraries, including ChemDiv, Mcule and LifeChemicals, are more structurally diverse for whether the Level 1 scaffolds or Murcko frameworks, and two libraries, including TCMCD and Specs, are less structurally diverse. But the quantity statistics cannot reveal similarities among these scaffolds, and the scaffolds of TCMCD may present more diverse in similarity. Besides, the exact trends of CSFPs for the Murcko frameworks and Level 1 scaffolds are also different. The CSFPs for the Murcko frameworks are more discriminatory. It is possible that more granular Murcko frameworks enhance the apparent scaffold diversity. Moreover, PC50C is also just a simple index at a certain point in CSFPs. Therefore, a more comprehensive comparison within the distributions of the Level 1 scaffolds is necessary to evaluate the structural features of these libraries.

### Tree Maps

In the previous section, we analyzed the scaffold diversity of the 12 libraries using the distributions of molecules over scaffolds. Our analyses show that the studied libraries are not evenly distributed over scaffolds, but we know little about the structural similarity and distribution of representative scaffolds. Thus, Tree Maps was used to visualize the structural similarity and distribution of the Level 1 scaffolds.

**Fig. 6:**
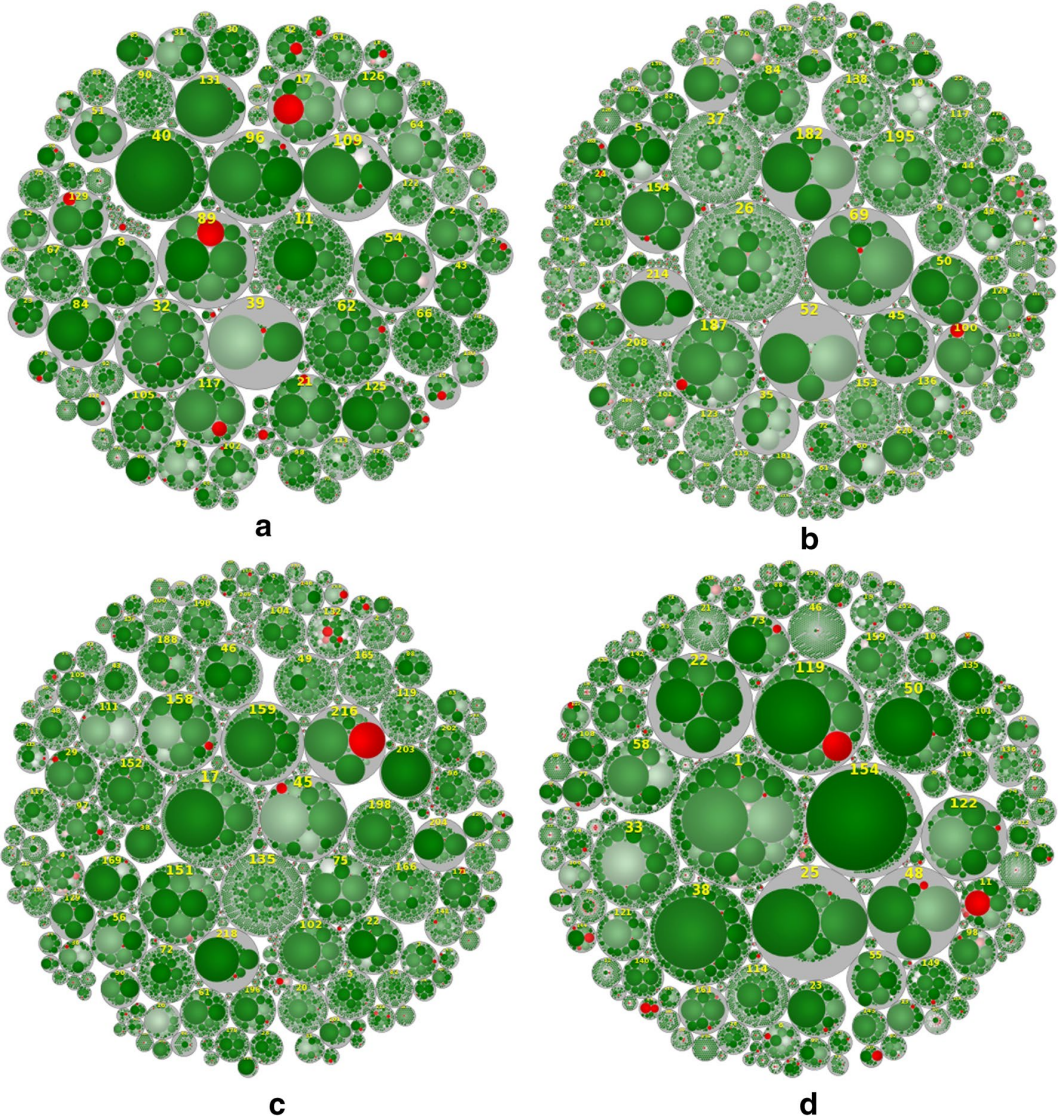
Tree Maps of the Level 1 Scaffolds for **a** LifeChemicals, **b** Enamine, **c** Mcule and **d** TCMCD

In Fig. 6 and Additional file 2: Fig. S1, colors in these circles are related to DistanceToClosest (DTC). That is to say, the deeper the red color is, the more similar the scaffold will be to the cluster center, and on the contrary, the deeper the green color is, the more dissimilar the fragment will be to the cluster center. As observed in those 12 Tree Maps, green, especially deep green, accounts for large areas in most of the datasets. To describe it easier, the deep green coverage ratio is defined as “Forest Coverage” (FC). As shown in Fig. 6, the FC values of TCMCD and LifeChemicals are larger than those of Enamine and Mcule, indicating that the Level 1 scaffolds in every gray circle of Enamine and Mcule are more similar to each other than those of the other two datasets. This is consistent with the results reported by Yongye et al. that natural products showed low molecule overlap [37]. Nevertheless, in a whole view, the separate gray circles for TCMCD and LifeChemicals are sparser than those for Enamine and Mcule, suggesting that the Level 1 scaffolds of Enamine and Mcule own higher structural diversity than the others. This is also demonstrated by the cluster numbers of Enamine, Mcule, TCMCD and LifeChemicals, which are 226, 220, 162 and 131, respectively. According to the analysis of CSFPs, it is believed that Enamine and Mcule may be more structurally diverse, which may result from more clusters not more diversity in similarities among molecular structures. By contrast, in LifeChemicals, however, despite some high dissimilarity appears in some clusters, these dissimilarities centralize in several kinds of scaffolds, resulting in much less unique fragments.

**Fig. 7:**
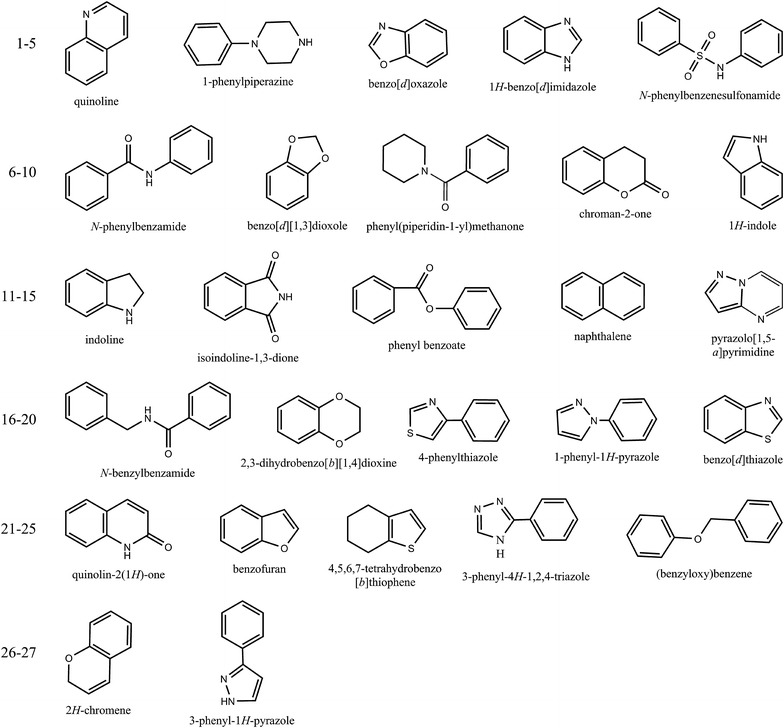
The Level 1 scaffolds with frequencies ≥2 found in the 10 most frequently occurring scaffolds (*1*–*25*) and the 10 scaffolds of the cluster centers in the top 10 clusters (*26*–*27*)

In order to compare the difference of the representative structures identified in the studied libraries, the 10 most frequently occurring scaffolds and the 10 scaffolds of the cluster centers in the top 10 clusters of each library were extracted (Additional file 2: Figs. S2, S3) and these two kinds of extracted scaffolds were merged respectively. Then, the frequencies of the merged scaffolds were counted and the scaffolds with frequencies ≥2 are shown in Fig. 7. Frequencies of these scaffolds for No. 1, 2, 4, 6 and 7 fragments found in different datasets are over 5. Interestingly, 8 out of the 10 most frequently occurring scaffolds of TCMCD cannot be found in any of the other 11 libraries. They have many non-aromatic rings with less nitrogen and more oxygen, and are quite different from the scaffolds found in the other libraries. By contrast, commercial libraries (except Maybridge) possess many common frequently occurring scaffolds with frequencies higher than 5.

In Additional file 2: Fig. S3, these scaffolds acting as the cluster centers in Tree Maps are obviously more dissimilar between each other. As shown in Fig. 7, there are only 2 scaffolds (26 and 27) with frequencies ≥2, which can be found in ChemBridge and LifeChemicals, and ChemDive and Maybridge, respectively. It seems that the scaffolds of these cluster centers serving as the representatives for clusters are more unique than the most frequent ones.

### SAR Maps

In the previous two sections, the structural features, distributions and scaffold diversity of 12 libraries have been analyzed, but the relationships among the scaffolds present in clusters for different libraries have not been explored. Then, the chemical space of the molecules identified by the substructure search of the representative scaffolds, which are the cluster centers from the Tree Maps for the 12 subsets, was characterized by the SAR Maps methodology. Besides, high interests in diverse scaffolds that preferentially interact with important target families are also taken into consideration [38]. The underlying pharmacological characteristics of some representative scaffolds which are important components of drug candidates against different drug targets are also predicted.

**Fig. 8:**
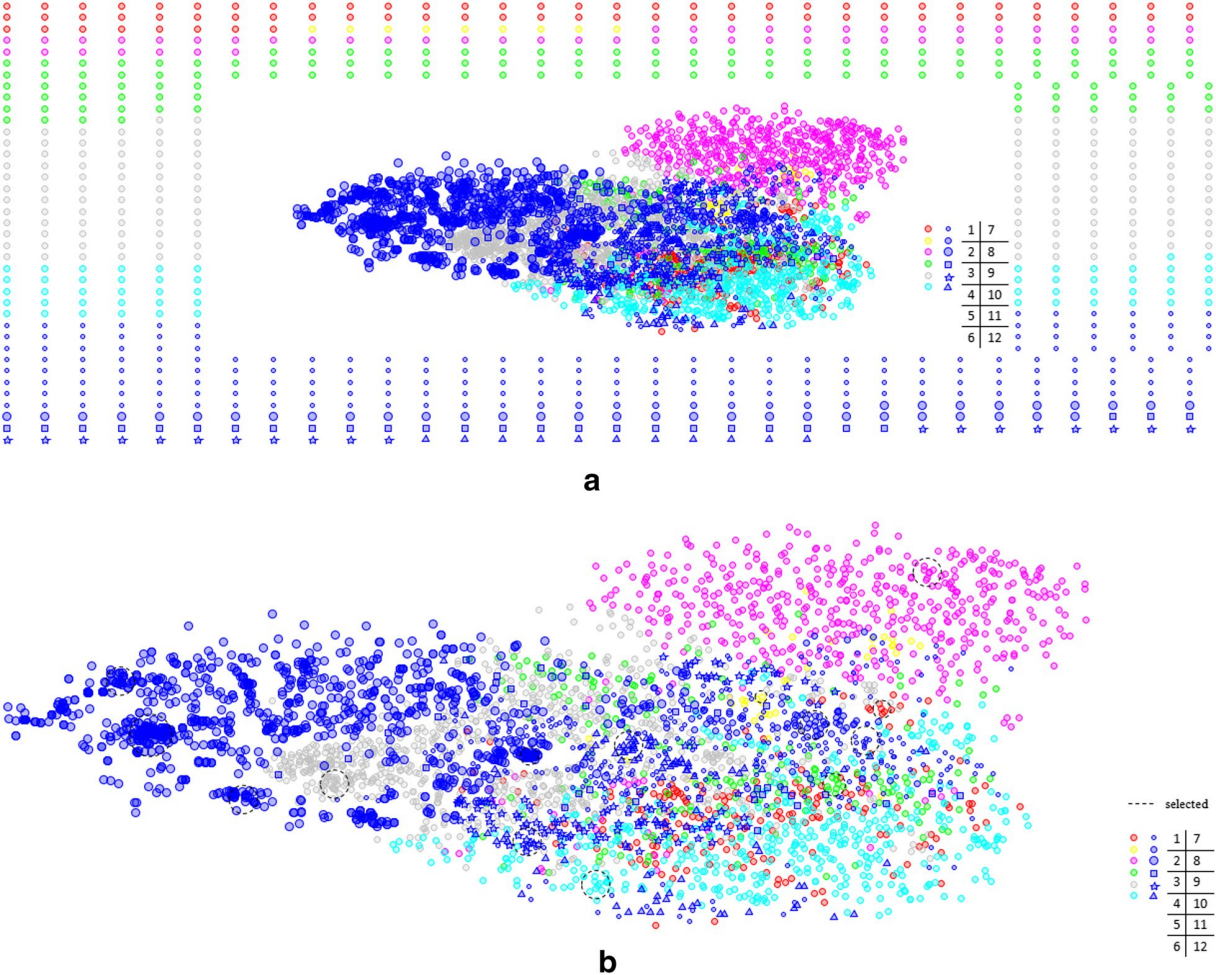
**a** The panoramic SAR Map of the Level 1 scaffolds for the 12 datasets. The numbers of molecules for *1* ChemBridge (260), *2* ChemDiv (47), *3* ChemicalBlock (562), *4* Enamine (328), *5* LifeChemicals (900), *6* Maybridge (513), *7* Mcule (518), *8* Specs (106), *9* TCMCD (1268), *10* UORSY (62), *11* VitasM (140) and *12* ZelinskyInstitute (112); **b** the center part of the SAR Map, and some selected groups of the representative molecules (39 in total) are highlighted by the *black dotted lines*

As shown in Fig. 8a, each point represents a molecule, and therefore, there are 4816 molecules in the SAR map. As mentioned above, points around the edge of the map are the molecules whose dissimilarities measured by the Tanimoto distance with all the others are higher than 0.3. Two molecules will be placed far apart if their similarity is < 0.3. It should be noted that it is meaningless to compare the molecules at the hedge with any other points in the map in terms of the actual similarity. More compounds on the hedge, such as Enamine (green circles), LifeChemicals (gray circles) and Mcule (the smallest blue circles), may imply higher structural diversity of the representative molecules. It is more meaningful to examine the points that are put together as the typical molecules in these libraries.

Therefore, to focus on the gathered molecules, the original SAR Map is magnified and shown in Fig. 8b. Compounds in the same library are represented by the points with the same color, size and shape. As shown in Fig. 8b, most of the biggest blue circles in TCMCD lie on the left of the map, and vast of the pink circles of ChemicalBlock on the upper right. Similarly, most light blue circles of Maybridge are at the bottom. As for the other libraries, such as Mcule represented by the smallest blue circles, it distributes more sparsely with few dense parts. But Mcule has 518 representative molecules, roughly equal to that of Maybridge (513) on the map. More dispersive distribution of Mcule suggests that Mcule also owns a large number of diverse molecules. The gray ones of LifeChemicals also spread in a wide range, but some accumulate in certain separated areas. Thus, there must be some distinct molecules in each library as shown by the denser areas on the map. Then, 40 selected areas of representative molecules highlighted by the black dotted circles on the SAR Map were identified.

To grasp the potential functions and structural properties of these selected representative molecules, similarity searching and the MCS searching were carried out. By searching BindingDB based on similarity, similar inhibitors of the representative molecules and the corresponding targets were obtained. Similar molecules in BindingDB could be found for 39 out of the 40 representative molecules, and the 39 corresponding MCSs are shown in Additional file 2: Fig. S4 and the potential targets are listed in Additional file 2: Table S1. We found that many identified potential targets were kinases and GPCRs with high similarity thresholds, such as Pyruvate kinase for ChemDiv, streptokinase A precursor for ChemicalBlock, Cyclin-Dependent kinase for LifeChemicals, Serine/threonine-protein kinase for Maybridge, hexokinase and Serine-protein kinase for TCMCD and Glycogen synthase kinase for LifeChemicals, Maybridge, Mcule, TCMCD, VitasM and ZelinskyInstitute. Moreover, GPCRs were also identified as the potential targets for the representative molecules found in ChemBridge, ChemicalBlock, Maybridge, TCMCD and VitasM. In particular, three groups of molecules in TCMCD have high similarity (up to 1) to the inhibitors of GPCRs but MCSs of the representative structures from these groups are not that similar. Besides, some ion channels, transporters, etc. can also be found as the potential targets. Our results suggest that these typical structures found by the SAR Maps can reveal some important structural and potential functional features for each dataset. Specifically, TCMCD, ChemicalBlock and Maybridge occupying unique area in chemical space, are of great potential to find drug candidates of those vital druggable targets, such as kinases and GPCRs.

## Conclusions

In this study, based on seven different fragment representations, the structural features, scaffold diversity and chemical distributions of 12 libraries, including 11 commercially available compound libraries and TCMCD, were explored and compared. The analyses indicate that although Chembridge, ChemicalBlock, Mcule, TCMCD and VitasM are more structurally diverse than the other databases. TCMCD is actually not quite structurally diverse for simple molecules, but the most occurring Level 1 scaffolds of it has tremendous difference to those of the other libraries. Despite Chembridge, Mcule and VitasM are rich in different kinds of fragments, their representative molecules largely overlap with those of the other databases, suggesting that the unique compounds in these libraries may be not so high in fact. Structures in ChemicalBlock are really diverse and complicated enough for VS. As for LifeChemicals, it does not have a variety of fragments but has much dissimilar molecular structures. Some libraries such as Enamine and UORSY are not good choice for actual VS considering the structural complexity and diversity of the molecules. Besides, 40 groups of representative scaffolds were identified in these 12 databases through Tree Maps and SAR Maps, and some molecules with these representative scaffolds found in certain libraries may be potential inhibitors of kinases and GPCRs. We believe that our study may provide valuable information to select proper commercial libraries in practical VS.

## Additional files

**Additional file 1: File S1.** Studied structures in the SAR map.zip.

**Additional file 2: Fig. S1.** Tree Maps for the studied datasets; **Figure S2.** The most frequent scaffolds served as the representatives of the clusters; **Figure S3.** Cluster centers served as the representatives of the clusters; **Figure S4.** Maximum Common Substructures (MCS) for 39 out of the 40 representative molecules; **Table S1.** Potential targets of the 39 similar molecules found in BindingDB for the 40 representative molecules.
