# Supplementary material for: Comparative analyses of structural features and scaffold diversity for purchasable compound libraries
Source: J Cheminform. 2017 Apr 21;9:25. doi: 10.1186/s13321-017-0212-4 (PMC5400773; doi:10.1186/s13321-017-0212-4)
Supplement: Supplementary file 2 — Additional file 2: Fig. S1. Tree Maps for the studied datasets; Figure S2. The most frequent scaffolds served as the representatives of the clusters; Figure S3. Cluster centers served as the representatives of the clusters; Figure S4. Maximum Common Substructures (MCS) for 39 out of the 40 representative molecules; Table S1. Potential targets of the 39 similar molecules found in BindingDB for the 40 representative molecules. [file 13321_2017_212_MOESM2_ESM.docx]

**Supporting Information**


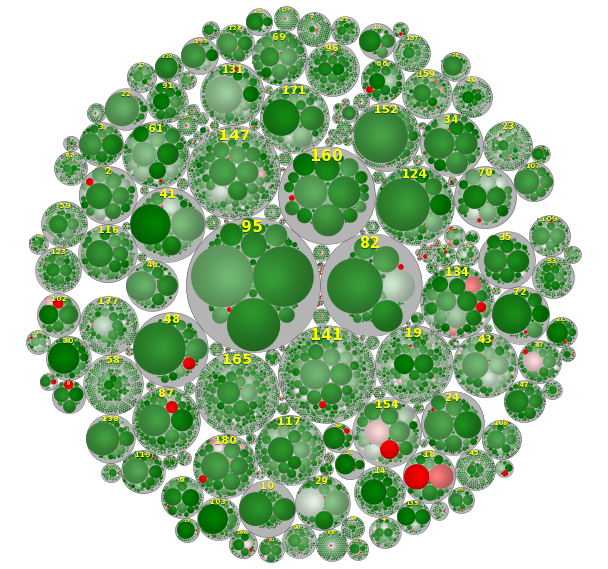


(1) Tree Map of ChemBridge

.
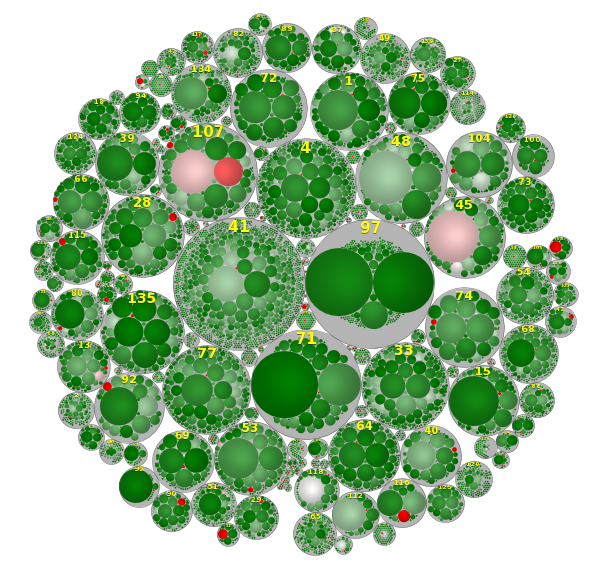


(2) Tree Map of ChemDiv.


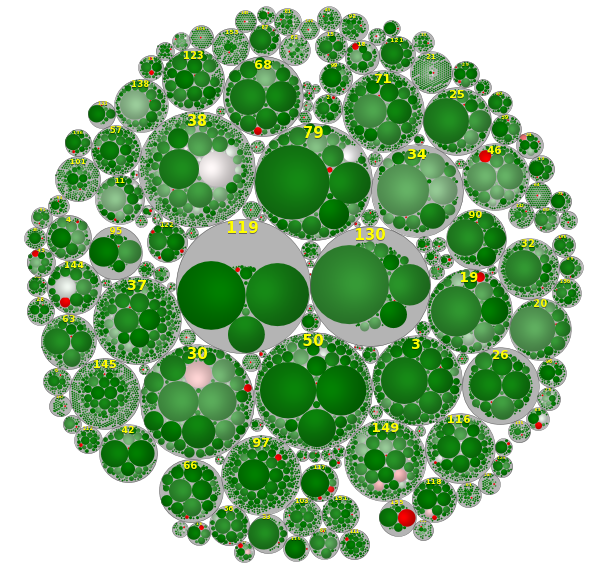


(3) Tree Map of ChemicalBlock.


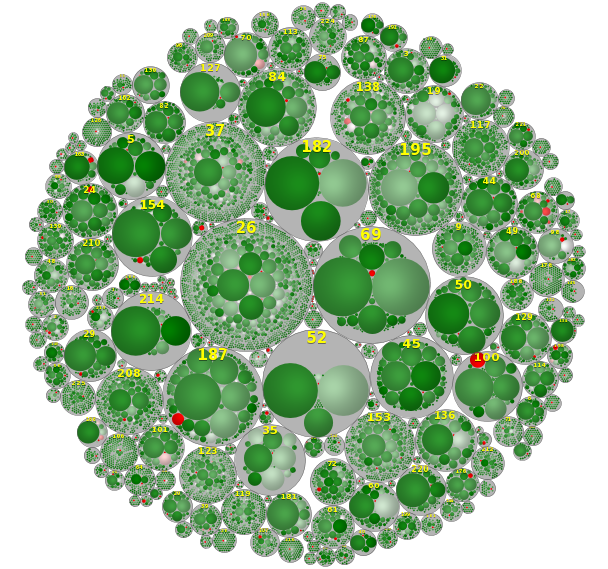


(4) Tree Map of Enamine.


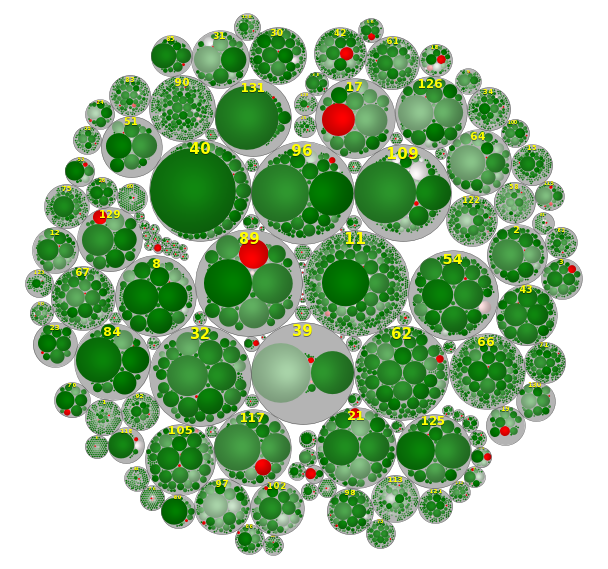


(5) Tree Map of LifeChemicals.


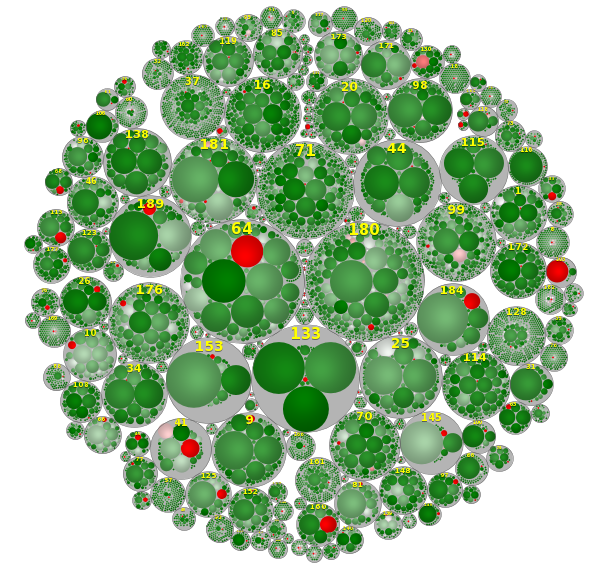


(6) Tree Map of Maybridge.


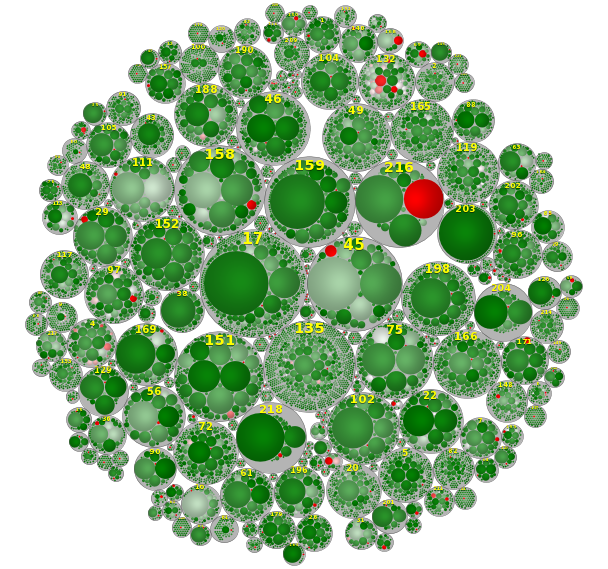


(7) Tree Map of Mcule.


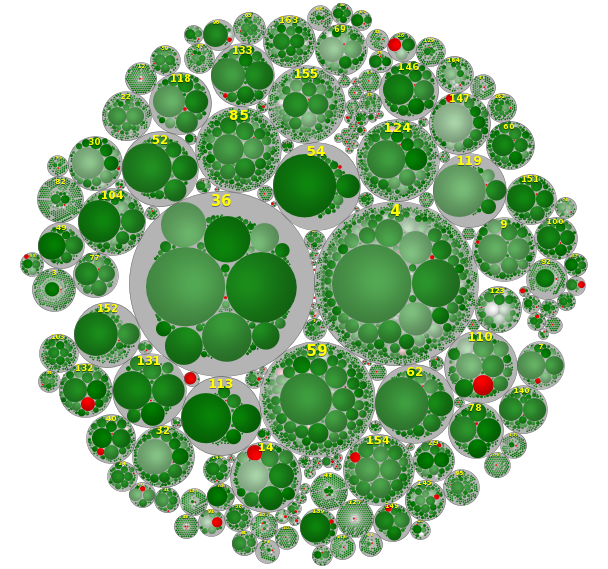


(8) Tree Map of Specs.


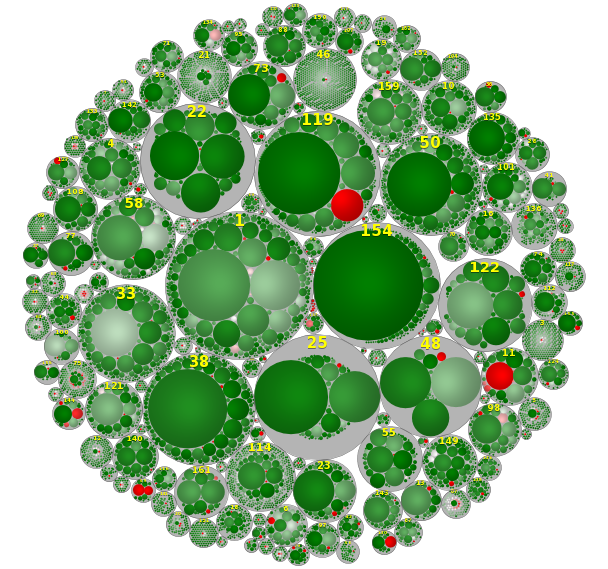


(9) Tree Map of TCMCD.


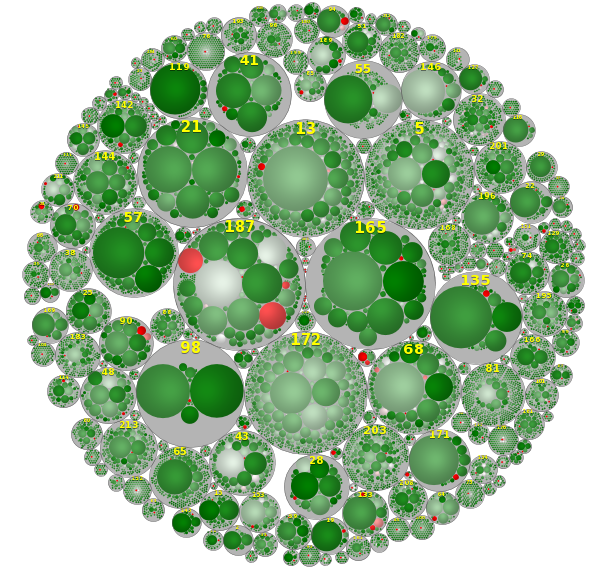


(10) Tree Map of UORSY.


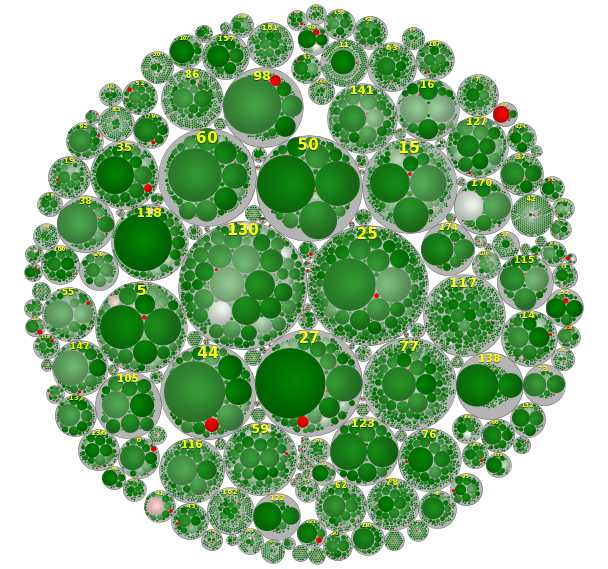


(11) Tree Map of VitasM.


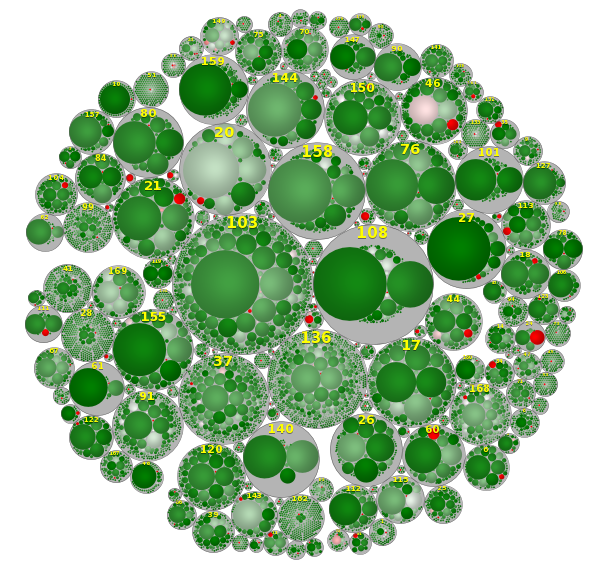


(12) Tree Map of ZelinskyInstitute.

**Figure S1**. Tree Maps for the studied datasets.


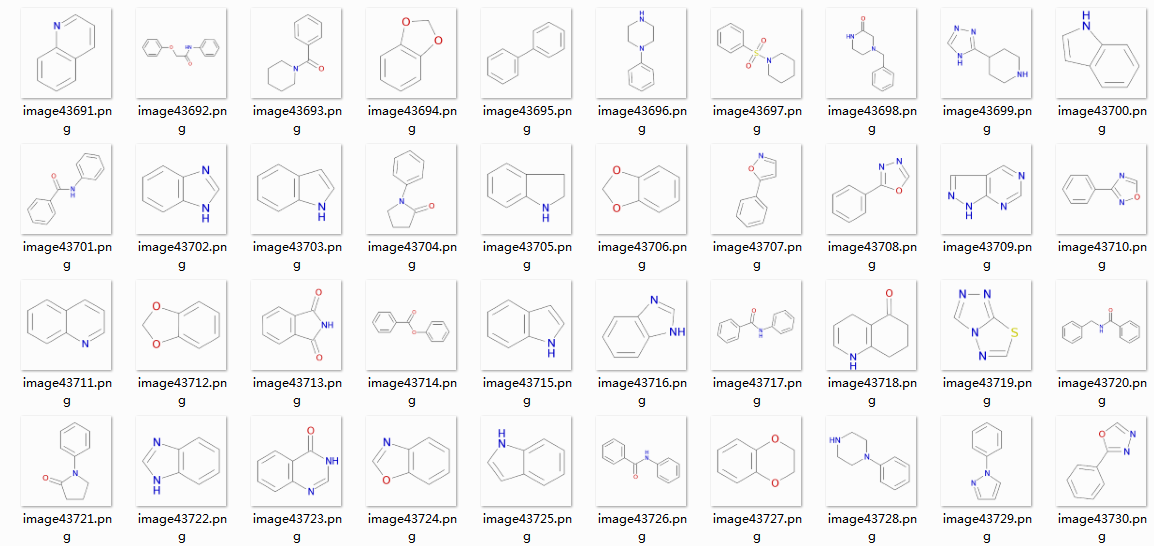


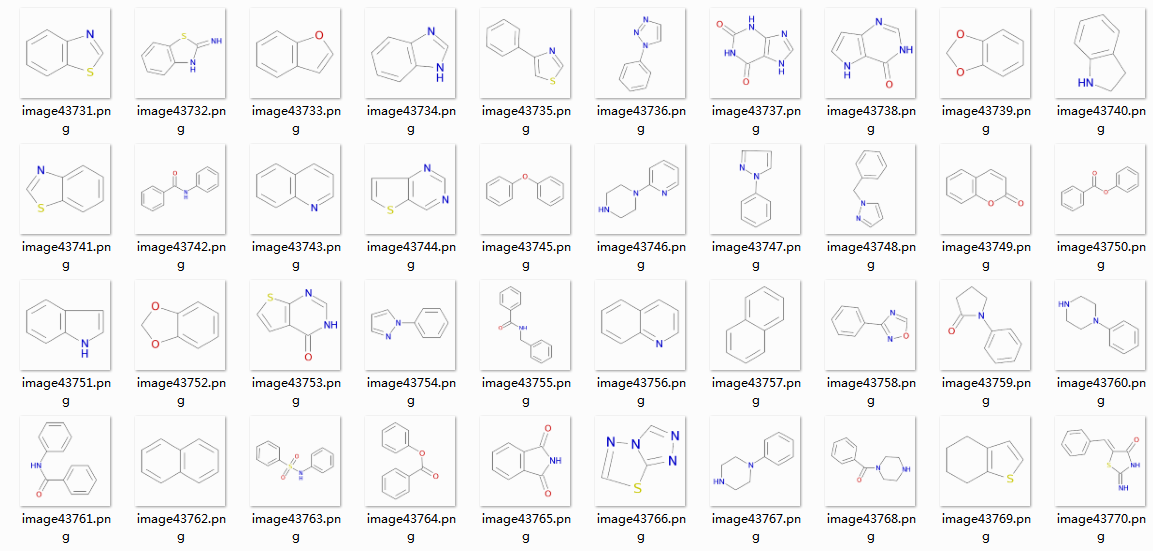
(a). From top to bottom, scaffolds of each line are from ChemBridge, ChemDiv, ChemicalBlock and Enamine, respectively.

(b) From top to bottom, scaffolds of each line are from LifeChemicals, Maybridge, Mcule and Specs, respectively.


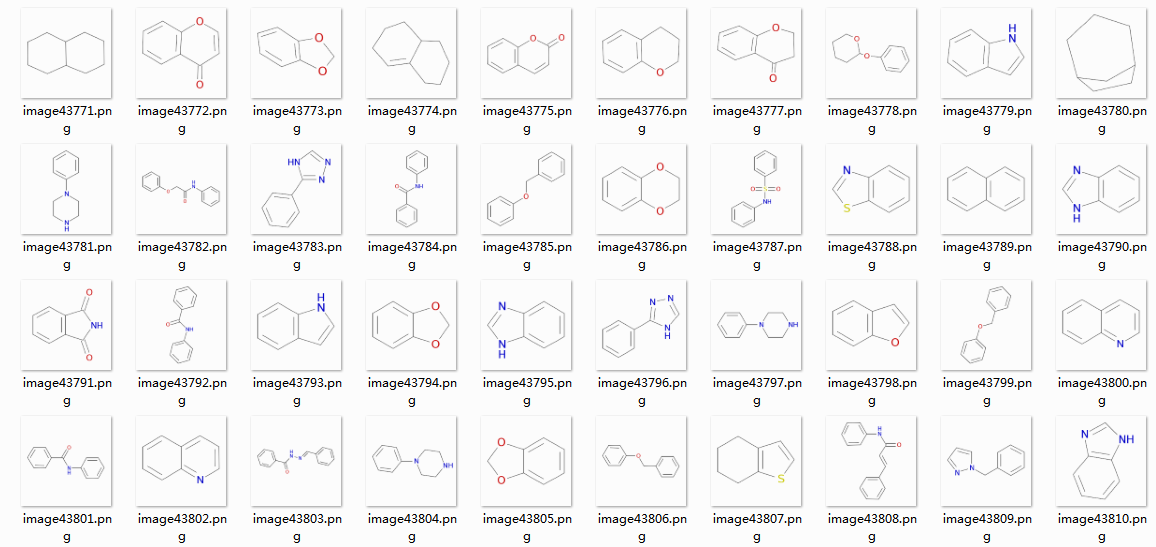


(c) From top to bottom, scaffolds of each line are from LifeChemicals, Maybridge, Mcule and Specs, respectively.

**Figure S2**. The most frequent scaffolds served as the representatives of the clusters


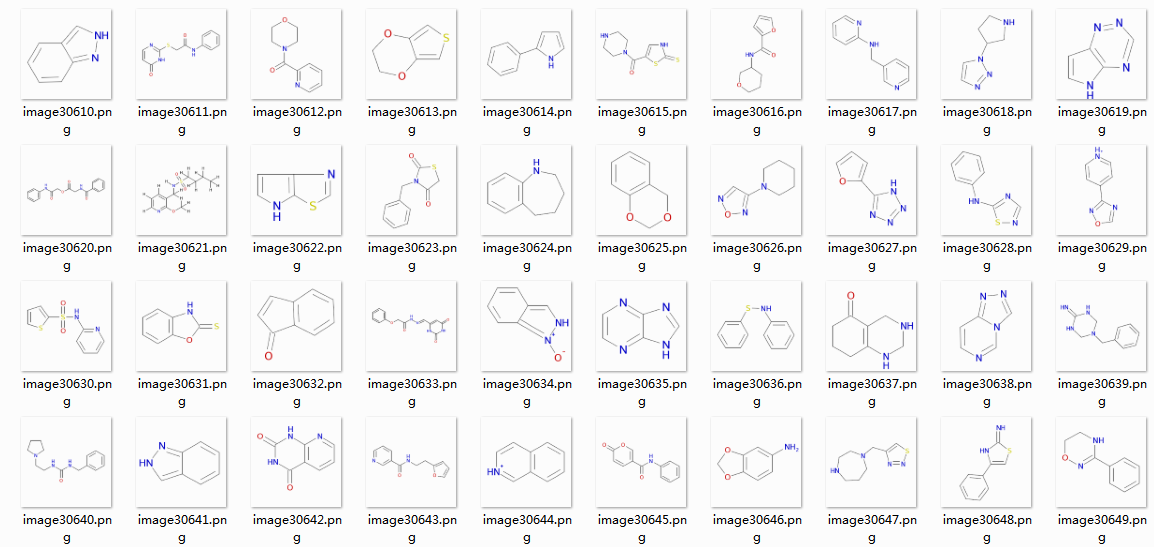


(a) From top to bottom, scaffolds of each line are from ChemBridge, ChemDiv, ChemicalBlock and Enamine, respectively.


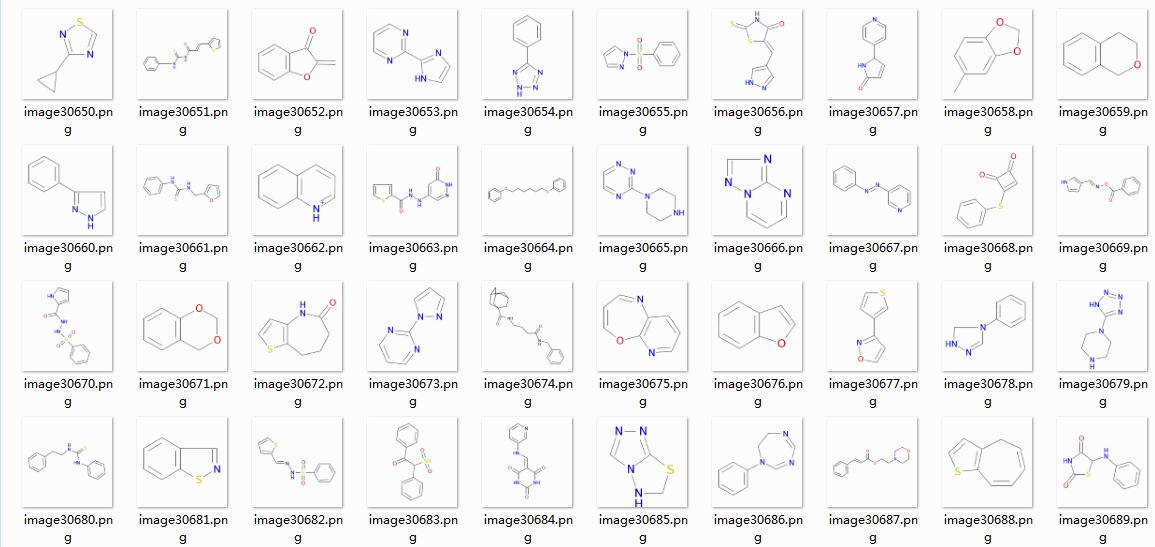


(b) From top to bottom, scaffolds of each line are from LifeChemicals, Maybridge, Mcule and Specs, respectively.


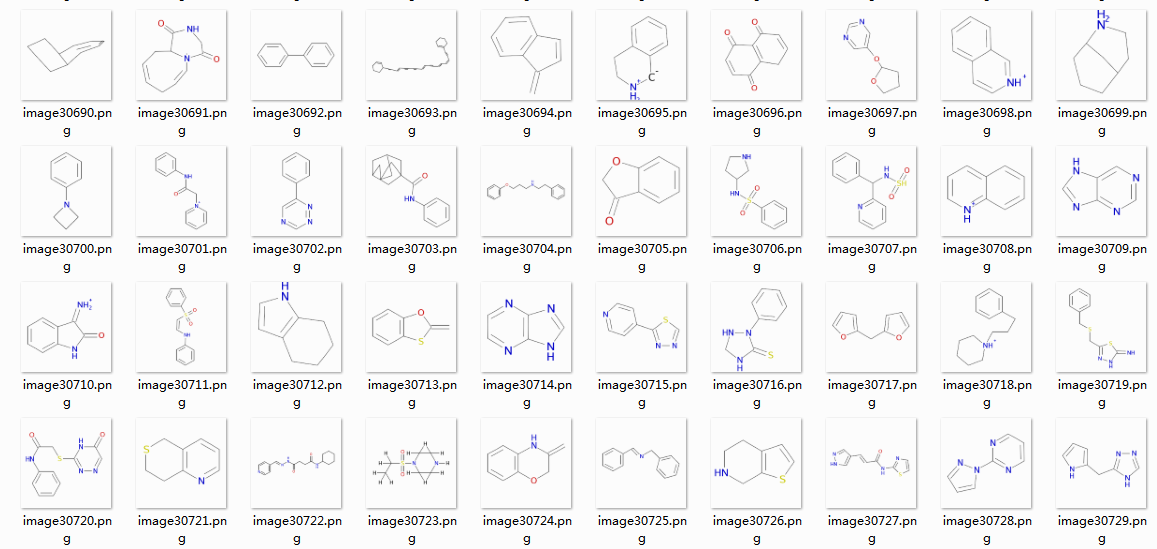


(c) From top to bottom, scaffolds of each line are from TCMCD, UORSY, VitasM and ZelinskyInstitute, respectively.

**Figure S3**. Cluster centers served as the representatives of the clusters.


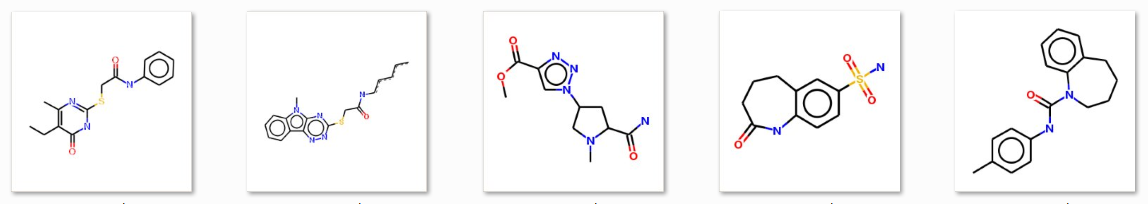

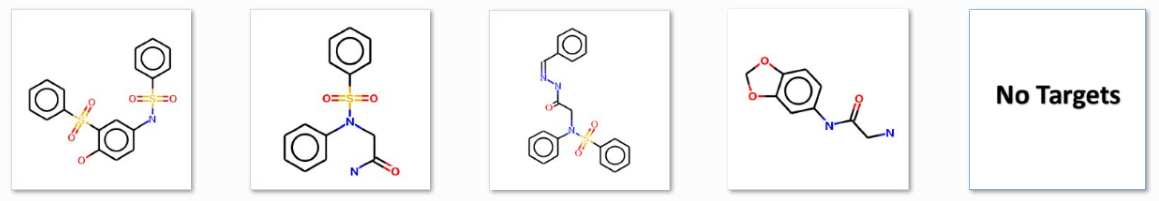

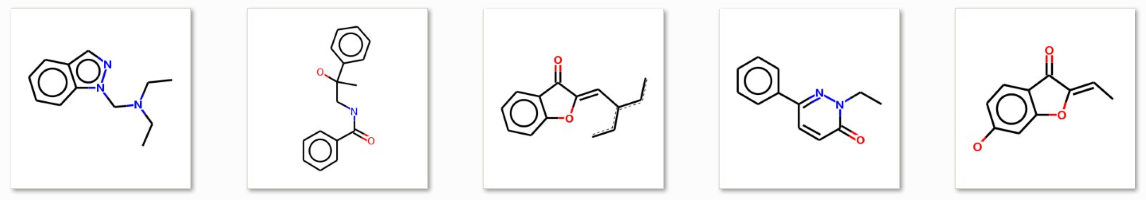

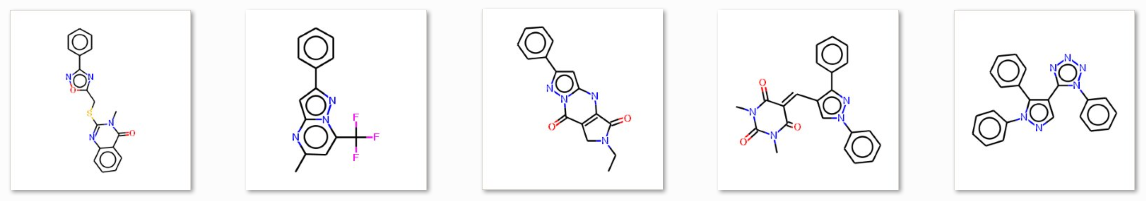

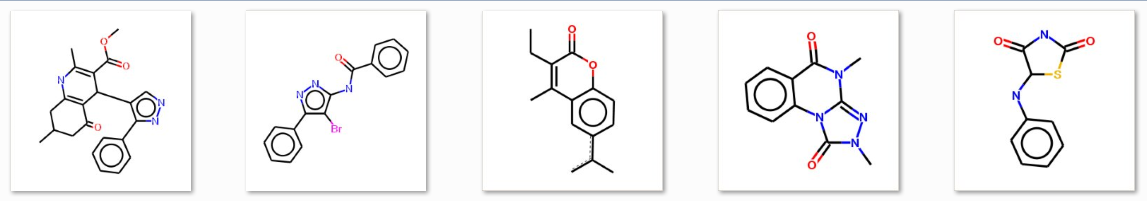

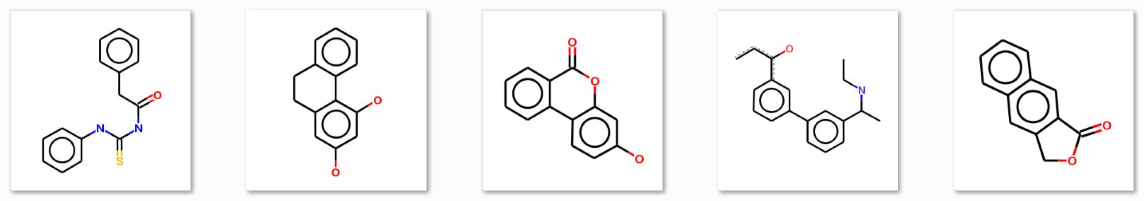

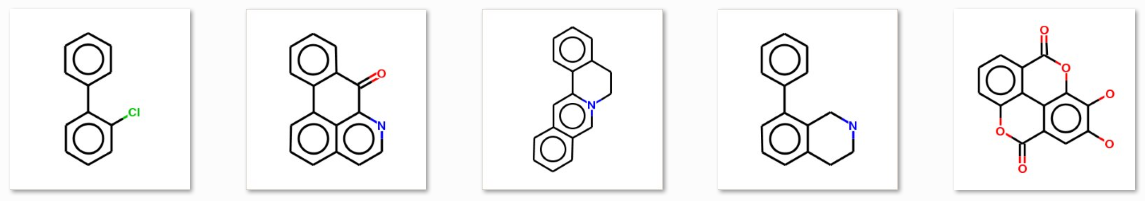

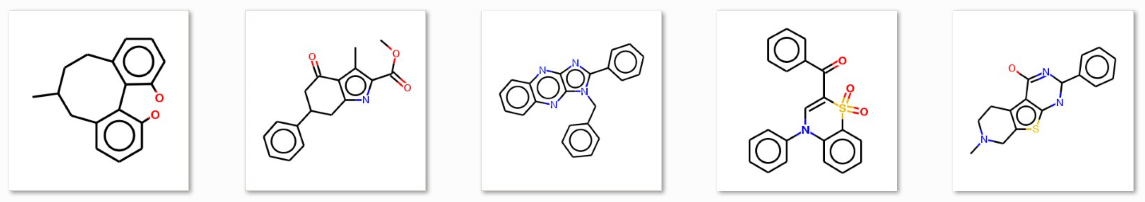
Note: Every maximum common substructure (MCS) is obtained from a group of molecules, which is one of the 40 groups as the queries in similarity searching. The order of these MCSs is corresponding to that of the groups/rows in potential targets shown in Table S1 from left to right and from top to bottom. “No Targets” shown above is the tenth group of the queries without finding any similar molecules at a similarity threshold of 0.6.

**Figure S4**. Maximum Common Substructures (MCS) for 39 out of the 40 representative molecules.

**Table S1**. Potential Targets of the 39 similar molecules found in BindingDB for the 40 representative molecules

| **Databases** | **Target** | **Similarity** |
| --- | --- | --- |
| ChemBridge | nuclear receptor subfamily 5 group A member 2 isoform 2 leucine aminopeptidase Estrogen receptor transient receptor potential cation channel, subfamily N, member 1 MCOLN3 protein | 0.8+ |
|  | Acetylcholinesterase Zinc aminopeptidase Estrogen receptor protein tyrosine phosphatase Ephrin receptor 0.91 Hsf1 protein | 0.8 |
|  | Prolyl endopeptidase Dipeptidyl peptidase Seprase | 0.7 |
| ChemDiv | karyopherin Pyruvate kinase (PKM2) Carbonic Anhydrase IX | 0.85 |
|  | Vasopressin V1/2 Receptor | 0.78 |
| ChemicalBlock | streptokinase A precursor | 0.84 |
|  | Cannabinoid receptor Vasopressin V1 receptor 0.9+ Oxytocin receptor 0.9+ Orexin receptor 1/2 Ubiquitin C-terminal Hydrolase L3 (UCH-L3) corticotropin releasing factor-binding protein ATP-binding cassette, sub-family C, member 1 isoform 1 Hsf1 protein LANA | 0.9+ |
|  | Oxytocin receptor 0.95 Vasopressin V1 receptor Thrombin and coagulation factor VII Dopamine receptor D2L/neurotensin receptor NTS1 0.9 | 0.85+ |
| Enamine | Complement C1 | 0.93 |
|  | no | >=0.6 |
| LifeChemicals | Melanin-concentrating hormone receptor | 0.76 |
|  | Phospholipase C 0.97 POsterior Segregation family member (pos-1) 0.99 Glycogen synthase kinase-3 0.84 cardiac alpha tropomyosin 0.96 Kallikrein 7 0.98 | 0.9 |
|  | Aldose reductase 0.62-0.66 | 0.6 |
|  | Aldehyde oxidase (AO) Fructose-bisphosphate aldolase Mcl-1 Cyclin-Dependent Kinase 5 (CDK5) 0.95 Monoamine oxidase（MAO） Histone deacetylase（HDAC1/2） Alpi Thrombin Tyrosinase PSMD14 protein Alkaline phosphatase Intestinal alkaline phosphatase Acetylcholinesterase POsterior Segregation family member (pos-1) Glycogen synthase kinase-3 drug resistance protein 2 | 0.95+ |
|  | neutrophil cytosolic factor 1 0.71 | 0.8 |
| Maybridge | Estrogen receptor Protein DAF-12, isoform a 0.82 Liver X receptor beta (LXRB) 1-deoxy-D-xylulose-5-phosphate synthase | 0.8 |
|  | Serine/threonine-protein kinase Aurora-A large T antigen 0.85 | 0.8+ |
|  | Histone deacetylase 1 (HDAC1) 0.77 Voltage-gated calcium channel STAT3 Alkaline phosphatase placental-like Transcription Factor STAT1 Hsf1 protein Glycogen synthase kinase-3 Dopamine receptor D2L/neurotensin receptor NTS1 POsterior Segregation family member (pos-1) | 0.75 |
|  | Cannabinoid receptor Voltage-gated T-type calcium channel | 0.7 |
|  | cardiac alpha tropomyosin karyopherin alpha 2 (RAG cohort 1, importin alpha 1), isoform CRA_b | 0.7- |
|  | Neuropeptide Y receptor type 5 ( NPY Y5) Bradykinin B1 receptor 1.0 | 0.8 |
| Mcule | Glycogen synthase kinase-3 Nuclear factor NF-kappa-B complex Kallikrein 7 | 0.9+ |
|  | DNA dC->dU-editing enzyme APOBEC-3G 0.88 | 0.8 |
| Specs | leucine aminopeptidase poly(A) binding protein, cytoplasmic 1 LANA 1.0 | 0.8+ |
|  | XBP1 neuropeptides B/W receptor 1 1.0 DNA damage-inducible transcript 3 protein C-C chemokine receptor type 6 (CCR6) Beta-galactosidase corticotropin releasing factor-binding protein | 0.8 |
| TCMCD | Tubulin 0.88 LXR-alpha 5-lipoxygenase/FLAP alpha-Glucosidase (alpha-Glu) Liver X receptor beta (LXRB) | 0.8 |
|  | Carbonic Anhydrase IX Zn finger protein Carbonic Anhydrase XII/13（1.0）/2/1/6/7/VA Aldehyde oxidase (AO) | 0.85 |
|  | 12-Lipoxygenase (12-LOX) Arachidonate 15-lipoxygenase Phospho-N-acetylmuramoyl-pentapeptide-transferase 0.96 | 0.9+ |
|  | 5-lipoxygenase/FLAP cGMP-specific 3',5'-cyclic phosphodiesterase 1.0 Phosphodiesterase 3 0.99 nuclear receptor subfamily 0 group B member 1、Steroidogenic Factor 1、Glycogen synthase kinase-3、FXN frataxin 1.0 | 0.85 |
|  | Cytochrome P450 1B1 0.88 Aryl hydrocarbon receptor | 0.9 |
|  | Acetylcholinesterase Cholinesterase defective in Germ Line Development family member (gld-1) 1.0 putative hexokinase HKDC1 1.0 Dopamine receptor D1、Dopamine Transporter (DAT) 0.82 Dopamine D2 receptor and Serotonin 2a receptor (D2 and 5HT2a)/D1 1.0 Human immunodeficiency virus type 1 REV 1.0 Protein tyrosine phosphatase receptor type C-associated protein 1.0 POsterior Segregation family member (pos-1) 1.0 | 0.75+ |
|  | Acetylcholinesterase Cholinesterase P2X purinoceptor 7 (P2X7R) Butyrylcholinesterase BZLF2 ubiquitin-conjugating enzyme E2I SUMO/sentrin specific peptidase family member 8 Neuraminidase Telomerase reverse transcriptase Voltage-gated potassium channel | 0.9 |
|  | Alpha adrenergic receptor (1a and 1d) Serotonin (5-HT) receptor Serotonin transporters (SERT) Dopamine receptor（DRD1/2/3） Histamine H2 Receptor Kappa/Delta Opioid Receptor Muscarinic acetylcholine receptor M1 and M3/2/4 Norepinephrine transporters (NET) Dopamine Transporter (DAT) | 0.9 |
|  | Carbonic Anhydrase1/2/7/9/12/13 DNA (cytosine-5)-methyltransferase 1 0.79 | 0.8 |
|  | Cytochrome P450 3A Serine-protein kinase ATR, Serine-protein kinase ATM, DNA-PK 0.87 Kappa Opioid Receptor 1.0 | 0.8+ |
| UORSY | too sparse to select a dense group of molecules from this database |  |
| VitasM | Protein Wnt-3a, Zinc finger protein GLI1, Nuclear receptor ROR-alpha, Steroidogenic Factor 1, COUP transcription factor 2 isoform a 0.97 nucleotide-binding oligomerization domain containing 2 Tumor necrosis factor (TNF-alpha) Sphingosine 1-Phosphate Receptor 3 Dopamine D2 receptor and serotonin 1a receptor, Serotonin (5-HT) receptor, Sphingosine 1-Phosphate Receptor 3/1, Alpha trans-inducing protein (VP16) 0.97 Factor XIIa (fXIIa) Liver X receptor beta (LXRB) NCOA2 protein, nuclear receptor coactivator 3 isoform a 0.96 Glycogen synthase kinase-3（GSK3） ATP-binding cassette, sub-family C, member 1 isoform 1 | 0.9 |
|  | cardiac alpha tropomyosin 0.89 | 0.9 |
|  | Hsf1 protein | 0.62 |
| ZelinskyInstitute | Integrase Cathepsin L1 protein tyrosine phosphatase, non-receptor type 7 isoform 2 corticotropin releasing factor-binding protein POsterior Segregation family member (pos-1) Bcl-2-like protein 11 Glycogen synthase kinase-3 | 0.85 |
| **Note:** |  |  |
| **Similarity** | The similarity threshold when searching molecules by a group of typical compounds found by SAR Map. |  |
| **decimal** | This can be found in the column of "target", which means the similarity between the searched and original molecules. The targets delimited by "," share a same exact similarity in the decimal form after them. |  |
| **Highlighted** | These targets of GPCR and kinases found in the search were colored in blue and red respectively. |  |
| **Target** | Every single receptor targeted by different molecules is delimited by space. Thoes targets delimited by caesura sign shared the same similarity threshold right after them in the blank of Target. |  |
| **Row** | Each row stands for a group of molecules found on the SAR Map as queries in similarity searching. |  |
